# Phylogenomic analysis of *Bupleurum* in Western Sichuan, China, including an overlooked new species

**DOI:** 10.3389/fpls.2023.1294670

**Published:** 2023-11-27

**Authors:** Zhi Chao, Xiaoxi Hong, Xuena Xie, Rong Huang, Enwei Tian

**Affiliations:** ^1^Department of Pharmacy, Zhujiang Hospital, Southern Medical University, Guangzhou, China; ^2^Faculty of Medicinal Plant and Pharmacognosy, School of Traditional Chinese Medicine, Southern Medical University, Guangzhou, China; ^3^Guangdong Provincial Key Laboratory of Chinese Medicine Pharmaceutics, Guangzhou, China

**Keywords:** Bupleurum, Western Sichuan, chloroplast genome, nuclear ribosomal DNA, phylogenomic analysis, Bupleurum pseudochaishoui Z. Chao sp. nov.

## Abstract

A comparative analysis of chloroplast (cp) genomes and 45s nuclear ribosomal DNA (nrDNA), and a phylogenomic study of six closely related species (including an overlooked new species) of genus *Bupleurum* from the western part of Sichuan Province in southwestern China were performed. The six species are similar morphologically and it is difficult to identify them; moreover, their genetic relationships remain unclear. It was found that the cp genomes of the six *Bupleurum* species were extremely similar, and they were highly homogeneous in terms of cp genome structure, genes and its arrangement. Intergenic spacer *rpl*32-*trn*L, *pet*A-*psb*J, *trn*K-*rps*16, and the coding gene *ycf*1 were considered highly variable. In phylogenetic trees constructed based on the complete cp genome, protein-coding sequences, nrDNA and ITS sequences, Chinese *Bupleurum* species all formed two major clades; among these trees, nrDNA tree had the best species resolution; the highly variable regions showed no advantage over other molecular markers. Among the six *Bupleurum* species, *B. malconense, B. sichuanense* were close relatives to *B. chinense* and *B. yinchowense*, *B. chaishoui* may also be a consanguinity, while *B. microcephalum, B. wenchuanense*, and the new species *B. pseudochaishoui* were closely related. At the end, the new species *B. pseudochaishoui* Z. Chao sp. nov. was described and illustrated, and a key to the six species was tabulated.

## Introduction

*Bupleurum* L., comprising about 210 species accepted nowadays (POWO, 2023), is one of the largest genera of the family Apiaceae. It has long been recognized as a natural group distinctively characterized by its members having simple leaves, mostly parallel veins, and conspicuous bracts/bracteoles. Infrageneric classification and phylogeny of the genus was of interest to plant taxonomists from the early days. The first use of section names in *Bupleurum* can be dated back to 1848 (Grenier and Godron, 1848). Wolff published a comprehensive revision of the genus that classified it into 5 sections (Wolff, 1910), and his classification is the most widely used for about a century. Shu et al. carried out a numerical taxonomic study in 1998 and divided the Chinese *Bupleurum* species into subgenus *Longifolia* and subgenus *Eubupleura* (including section *Ranunculoidea* and section *Falcata*) (Shu et al., 1998). Neves & Watson suggested dividing *Bupleurum* into two subgenera (subg. *Penninervia*, subg. *Bupleurum*) based on phylogenetic analyses of nuclear ribosomal internal transcribed spacer (nrITS) sequences of 35 taxa (Neves and Watson, 2004). Although the previous researches have deepened our understanding of the genus, it is still hard to say being sufficient.

China harbors abundant diversity of *Bupleurum*. In *Flora of China* published in 2005, 42 species, 16 varieties and 6 forms were recorded (She and Watson, 2005). After revisions of He et al. and Pimenov, etc. in recent years, it was acknowledged there are 47 species, 1 subspecies, 6 varieties and 5 forms of *Bupleurum* in China (Wang et al., 2011; Ma et al., 2013; Wang et al., 2013; Ma and He, 2015; Pimenov, 2017), most of which have reputed medicinal values in treating fever and liver ails (Liang et al., 2012; Chao et al., 2014), as observed in many wild species of Apiaceae (Moazzami Farida et al., 2018; Sonigra and Meena, 2021; Perrino et al, 2023).

In the western part of Sichuan Province in Southwestern China, i.e., some areas of Aba Zang and Qiang Autonomous Prefecture near Chengdu, including Wenchuan, Mao County, Songpan, and so on, the steep mountains and plateaus lead to the differentiation and formation of *Bupleurum* species (Wang et al., 2008a; Yang et al., 2021). Closely related species discovered and reported in this region include *B. wenchuanense* R.H. Shan & Y. Li, *B. chaishoui* R.H. Shan & M.L. Sheh, *B. malconense* R.H. Shan & Y. Li, *B. sichuanense* S.L. Pan & P.S. Hsu, *B. microcephalum* Diels, etc. (Pan and Hsu, 1992). Most of them are quite morphologically similar and the differences between each other are subtle, thus their identification is notoriously difficult. And also, their genetic relationship remains unclear.

Phylogenomics, especially that based on the whole chloroplast (cp) genome sequences, has been proved highly efficient in resolving complicated phylogenetic problems of taxonomically complexed plant groups (Liu et al., 2022; Zhang et al., 2023), and in differentiating and identifying closely related species (Pchelin et al., 2019; Li et al., 2021). We’ve made some efforts in elucidating phylogeny of Chinese *Bupleurum* through phylogenomic means. We sequenced cp genomes of two woody *Bupleurum* species (*B. gibraltaricum* Lam. and *B. fruticosum* L.) endemic to Mediterranean and made comparative analysis with some Chinese species, confirming the division of 2 subgenera (subg. *Penninervia*, subg. *Bupleurum*) and revealing their divergence time (Huang et al., 2021a); we also analyzed the sequences of whole cp genomes of 4 Southwest China endemic alpine *Bupleurum* species and suggested phylogenetic affinity in the genus might be closely related to geographical distribution (Huang et al., 2021b); and we proposed the phylogenetic position of *B. sikangense* C.B. Wang, X.G. Ma & X.J. He as well (Xie et al., 2021). As a continuation of the work, we made a phylogenomic analysis of the above-mentioned species distributed in western Sichuan, based on both cp genome and 45s nuclear ribosomal DNA (nrDNA, including 16s rRNA-ITS1-5.8s rRNA-ITS2-26s rRNA), in order to untangle the complicated relationship among them, and to seek suitable molecular markers for species identification.

Additionally, in the ongoing study on *Bupleurum* investigation, the authors encountered a population in which the individuals obviously belonged to this genus in Wenchuan county years ago. For all the time, the authors had been regarding the plants in this population as *B. chaishoui*. Until recently, the authors obtained some specimens of *B. chaishoui* in its type locality and had a deeper understanding of the species. The authors realized that the plants met before is unique in its leathery dimorphic leaves and represent an overlooked new species, and then named it as *B. pseudochaishoui*. It was thus described here.

## Materials and methods

### Plant materials

In this study, 65 plant samples, representing 29 *Bupleurum* species were collected in the summer of the years of 2014 through 2020, from North, Northeastern, Northwestern and Southwestern China, including 20 samples of the six above-mentioned species in Western Sichuan (**Table 1**). The voucher specimens were deposited in the herbarium of the University (Southern Medical University herbarium, SMU, to be listed in the Index Herbarium).

**Table 1:** Sources of material and GenBank accession numbers.

Taxa	Locality	Voucher	Accession number		
			cp genome	nrDNA	ITS
*Bupleurum pseudochaishoui* Z. Chao sp. nov.	Wenchuan. Sichuan	Chao Z 1682502	OQ460239	OQ627439	OQ506349
	Wenchuan. Sichuan	Chao Z 2020080849	OQ460238	OQ627450	OQ651273
	Wenchuan. Sichuan	Liuli 0908102			JF757223
*B. chaishoui* R.H. Shan & M.L. Sheh	Songpan, Sichuan	Chao Z 1682703	OQ460228	OQ627440	OP433488
	Maoxian, Sichuan	Chao Z 1683102	OQ460233	OQ627442	OP433487
	Maoxian, Sichuan	Chao Z 1773003	OQ460227	OQ627445	OP433486
	Heishui, Sichuan	Zhang DJ et al., s.n.			OP433484*
	Maoxian, Sichuan	Yuan CQ & Wang NH 92043			OP433489*
*B. malconense* R.H. Shan & Y. Li	Danba, Sichuan	Chao Z 1780601			OP433483*
	Deqin, Yunnan	Chao Z 1782001			OP433482*
	Jinchuan, Sichuan	Chao Z & Huang R 2020080421	OQ460229	OQ627451	OP433478
	Jinchuan, Sichuan	Chao Z & Huang R 2020080422	OQ460230	OQ627452	OP433481
	Rangtang, Sichuan	Ma XG 10091401			HQ687945
	Ma’erkang, Sichuan	Ma XG 09092103			GU269879
*B. microcephalum* Diels	Ma’erkang, Sichuan	Chao Z 1683101	OQ460232	OQ627441	OP433476
	Xiaojin, Sichuan	Chao Z 1780302			OP433474*
	Songpan, Sichuan	Chao Z 1773104	OQ460231	OQ627446	OP433473
	Lixian, Sichuan	Ma XG 090801403			GU269882
*B. sichuanense* S.L. Pan & P.S. Hsu	Wenchuan. Sichuan	Chao Z 1682501	OQ460234	OQ627438	OP433472
	Maoxian, Sichuan	Chao Z 1682601			OP433471*
	Maoxian, Sichuan	Chao Z 1773001	OQ460235	OQ627444	OP433470
	Wenchuan. Sichuan	Chao Z & Zhang DG 0308052			DQ285447
	Maoxian, Sichuan	Yuan CQ & Wang NH 92044			OP433477*
*B. wenchuanense* R.H. Shan & Y. Li	Wenchuan. Sichuan	Chao Z 1683103	OQ460237	OQ627443	OP433468
	Maoxian, Sichuan	Chao Z, Zhang DG 0308051			DQ285461
	Maoxian, Sichuan	Chao Z & Huang R 2020080639	OQ460236	OQ627449	
	Wenchuan. Sichuan	Ma XG 090831			HQ687964
*B. angustissimum* (Franch.) Kitag.	China	Wang H 00031289	MT534600		
	Wuqi, Shaanxi	Chao Z 1981601		OR493965	OR502915
	Jingbian, Shaanxi	Chao Z 1681802		OR493964	OR502914
	Shuozhou, Shanxi	Wang CB 09010			HQ824721
	Tongxin, Ningxia	Wang CB 09091			GU570631
*B. bicaule* Helm	China	Wang H 00031282	MT534603		
	Zalainuo'er, Neimenggu	Chao Z 2082302		OR493966	OR502916
	Chenba'erhu, Neimeng	Chao Z 2082401		OR493967	OR502917
	Neimenggu, China	Wang ZX 20100912			HQ824722
	Manzhouli, Neimenggu	Wang CB 09066			HQ824723
*B. boissieuanum* H. Wolff	China	zhc20111019-15-2	OQ621980		
	Taibai, Shaanxi	Chao Z 1680902	OR508810	OR493968	
	Chengkou, Chongqing	Chao Z 1882603	OR508811	OR493969	
	Kongtongshan, Gansu	Wang CB 09078-4			GU570624
	Taibaishan, Shaanxi	Wang CB 09132			HQ687905
*B. candollei* Wall. ex DC.	China	/	MT261183		
	Dali, Yunnan	Chao Z 1782501		OR493970	OR502917
	China	Xie H 040517002			DQ285471
	Kunming, Yunnan	Wang CB 20081105			GU269873
*B. chinense* DC.	Zhashui, Shaanxi	Zhang F 611026LY0126	MN337347		
	Feixi, Anhui	Chao Z 2171901	OR508812		
	Shexian, Heibei	Chao Z 2020081983	OR508815	OR493973	OR502921
	Shijiazhuang, Heibei	Chao Z 2020081458	OR508813	OR493971	OR502919
	Wuling, Beijing	Feng CQ 200508			EU001334
	Dabieshan, Anhui	Wang CB 09017			GU570615
*B. commelynoideum* H. Boissieu	Kangding, Sichuan	Chao Z 1682303	OR508818	OR493976	OR502924
	Kangding, Sichuan	Chao Z 1682201	OR508816	OR493974	OR502922
	Kangding, Sichuan	Chao Z 1682203	OR508817	OR493975	OR502923
	Kangding, Sichuan	Chao Z 1780605		OR493977	OR502925
	Kangding, Sichuan	Chao Z 2020080217		OR493978	OR502926
	Daocheng, Sichuan	Wang CB 09141			HQ687920
	Daofu, Sichuan	Ma XG 182			HQ687926
*B. densiflorum* Rupr.	China	/	MT261184		
	Tianshan, Xinjiang	Wang CB 09165			GU570611
*B. dracaenoides* Huan C. Wang, Z.R. He & H. Sun	China	/	MT387201		
	China	Wang HC 20110148			JQ365173
*B. euphorbioides* Nakai	Kangwon-do, Korea	Anonymous TKMII-33-2	MT821948		
	Changbaishan, Jilin	Wang CB 09038-5			GU570619
*B. falcatum* L.	Suihua, Heilongjiang	Zhang GX & Wang H 23000503	MT075716		
	South Korea	/	NC027834		
	Japan	Wei XQ 86			GU570605
	Lixian, Sichun	Ma XG 09092201			GU570639
	Hanyuan, Sichuan	Chao Z 2020072901	OR508820	OR493980	OR502928
	Xiangfen, Shanxi	Chao Z 2020081878	OR508821	OR493981	OR502929
	Toyama, Janpan	Chao Z 2009100601	OR508819	OR493979	OR502927
*B. fruticosum* L.	Cultivated, London	Chao Z 1871201	MW497417	OQ627447	OP433467
	Estremadura, Portugal	Neves 33			AF479297
*B. gibraltaricum* Lam.	Cultivated, London	Chao Z 1871202	MW497418	OQ627448	OP433466
	Sevilla, Spain	Neves 35			AF479851
	Sevilla, Spain	Neves & Watson 51			AF479852
*B. hamiltonii* N.P. Balakr.	Bijie, Guizhou	Anonymous HPCH0003	MW262986		
	China	Wang QZ & Pu FD 348618			EU001338
	Kunming, Yunnan	Wang CB 20081101			GU205475
	Huize, Yunnan	Chao Z 1782703		OR493982	OR502930
*B. kaoi* Liu, C.Y. Chao & Chuang	Miaoli, Taiwan	TAIE: 47911	OK050523		
	Taiwan	/			AM711598
*B. kweichowense* R.H. Shan	Jiangkou, Guizhou	Chao Z 1882001	MW135454	OR493983	OR502931
*B. latissimum* Nakai	South Korea	/	MT821949		
	Korea	/	NC 033346		
	South Korea	/			AY551292
*B. longiradiatum* Turcz.	Ningan, Heilongjiang	Chao Z 1481902	OR508823	OR493985	OR502933
	Tieli, Heilongjiang	Chao Z 1481601	OR508822	OR493984	OR502932
	Jiamusi, Heilongjiang	Wang CB 09034-1			GU570618
*B. marginatum*Wall. ex DC.	China	/	MT261187		
	Weishan, Yunnan	Wang ZX 2010091101			HQ687955
	Ranwu, Xizang	Chao Z 1781102	OR508826	OR493988	OR502936
*B. marginatum* var. *stenophyllum* (H. Wolff) R.H. Shan et Y.Li	Lintao, Gansu	Zhang GX 62000301	MT075712		
	Nielamu, Xizang	Yu Y 00107			HQ687952
	Huize, Yunnan	Chao Z 1782702	OR508825	OR493987	OR502935
	Jianshui, Yunnan	Chao Z 1782601	OR508824	OR493986	OR502934
*B. pusillum* Krylov	China	/	MT261188		
	Qinghai Lake, Qinghai	Ma XG 09151			HQ824728
*B. rockii* H. Wolff	Lijiang, Yunnan	Chao Z 1782301	MW135455	OR493989	OR502937
	Lijiang, Yunnan	TEW008	MW135456	OR493990	OR502938
*B. scorzonerifolium* Willd.	Lindian, Heilongjiang	Zhang GX & Wang H 23000403	MT075715		
	Zhangbei, Hebei	Anonymous, 130722LY0211	MT239475		GU570604
	Zibo, Shandong	Pang YL 200707			GU570604
	Chuzhou, Anhui	Wang ZX 2010071102			HQ687960
	E’erguna, Inner Mongolia	Chao Z 2082403		OR493991	OR502939
*B. shanianum* X.G. Ma & X.J. He	Baima Snow Mountain, Deqin, Yunnan	Chao Z 1782002	MW135452	OR493992	OR502940
*B. sibiricum* Vest ex Roem. & Schult.	China	/	MT261190		
	Daqingshan, Neimeng	Pan SL 0519			DQ285457
	Chifeng, Neimeng	Chao Z 1980701		OR493993	OR502941
*B. sikangense* X.J. He & C.B. Wang	Mangkang, Xizang	Chao Z 1780701	MW263066	OR493994	OR502942
	Mangkang, Xizang	Chao Z 1781901	MW263067	OR493995	OR502943
*B. smithii* H. Wolff	Guyuan, Ningxia	Chao Z 1582502	MN854381	OR493996	OR502944
	Wuzhong, Ningxia	Chao Z 1582702	MN854382	OR493997	OR502945
	Tianzhu, Gansu	Chao Z 2180101	OR508827	OR493998	OR502946
	China	Xie H 040727001			DQ285455
	Minhe, Qinghai	Ma XG 09080410			GU570637
*B. thianschanicum* Freyn	China	/	MT261192		
	China	Wang F 04-0111			EU220927
	Zhaosu, Xinjiang	Tuozi 780580			GU570608
*B. triradiatum* Adams ex Hoffm.	China	/	MT261193		
	Tianshan, Xinjiang	Chao Z 1573001		OR494000	OR502948
	Hami, Xinjiang	Anonymous 039			GU570635
*B. yinchowense* R.H. Shan & Y. Li	China	/	MT261194		
	Suide, Shanxi	Wang CB 09101			GU570629
	Jixian, Shaanxi	Wang CB 09008			HQ687966
	Yulin, Shaanxi	Chao Z 1681701	OR508829	OR494001	OR502949
	Zichang, Shaanxi	Chao Z 2280101	OR508830	OR494002	OR502950
	Jingbian, Shannxi	Chao Z 2280105	OR508831	OR494003	OR502951
*B. yunnanense* Franch.	Kangding, Sichuan	Chao Z 1682301	MW135450		
	Lijiang, Yunnan	Chao Z 1782303	MW135453	OR494004	OR502952
	Kunming, Yunnan	Wang QZ & Pu FD 374574			EU001349
	Shangri-la, Yunnan	Ma XG 10090302			HQ687969
*Apium graveolens* L.	Guangzhou, Guangdong	Chao Z 2019042602	OR508809	OR501374	OR502913
*Centella asiatica* (L.) Urb.	China	Chao Z 2019042601	MN854377	OR501375	OR502953
*Cryptotaenia japonica* Hassk.	Guangzhou, Guangdong	Chao Z 2019042603	OR508832	OR501376	OR502954
*Foeniculum vulgare* Mill.	Guangzhou, Guangdong	Chao Z 2019042604	OR508833	OR501377	OR502955

Sequences with accession number in bold face were obtained in this study; those marked with “*” were acquired through PCR amplification and Sanger sequencing. “/” means voucher specimen information unavailable.

Specimens of other Chinese *Bupleurum* species, especially those distributed in Western Sichuan, which were deposited in the major herbaria of China including China National Herbarium (PE), Herbarium of Department of Biology, Sichuan University (SZ), Herbarium of Chengdu Institute of Biology, Chinese Academy of Sciences (CDBI), Kunming Institute of Botany Herbarium (KUN) and Herbarium of College of Life Sciences, Northwest Agriculture & Forestry University (WUK), were also consulted and inspected on site or through Chinese Virtual Herbarium (CVH) website.

### Morphological inspection

The morphological characters of taxonomic significance in *Bupleurum* (Pan et al., 2002), i.e., the number, shape and size of bracteoles, comparison of bracteoles length and umbellule size, the number and length of rays, the number and size of florets, the shape, size and texture of leaves, of *B. pseudochaishoui* were inspected, and were compared with the related species, *B. wenchuanense*, *B. chaishoui*, *B. malconense*, *B. sichuanense*, and *B. microcephalum*. The data were acquired from at least 10 individuals of living materials or herbarium specimens, and were also checked in accordance with the descriptions in *Flora of China* (She and Watson, 2005). For measurements of some quantitative traits, such as plant height, leaf size, the number of small flowers, etc., 50 pieces of data were collected for each item, then the variation ranges were summarized and recorded.

### DNA extraction

Genomic DNA was isolated from the silica gel dried leaves of each sample using DNAsecure Plant Kit (TIANGEN Biotech Co, Ltd., Beijing, China). After purification, concentration of the extracted DNAs was determined on a NanoDrop 2000C spectrophotometry (Thermo Fisher, US).

### ITS region amplification and sequencing

ITS region of some samples (those with ITS accession number marked by “*” in **Table 1**) were amplified and sequenced. The universal primers ITS-p4 and ITS-p5 (Cheng et al., 2015) were used. PCR amplification was performed in a 15 μL reaction system containing 6 μL of 2×Taq Plus PCR MasterMix (Tiangen Biotech Co, Ltd., Beijing, China), 2 μL of DNA template, 0.5 μL of each primer (10 mM), and 6 μL of ddH_2_O. The PCR products were purified and sequenced bidirectionally by Ruibo Biotech Co. Ltd. (Guangzhou, China). The sequences have been deposited in GenBank, and the accession numbers were listed in **Table 1**. We also downloaded several ITS sequences of the related species from GenBank (**Table 1**).

### Skimmed genome sequencing

The purified genomic DNAs were subjected to genome skimming performed on an BGISEQ-500 sequencer by BGI Genomics (Shenzhen, China). For each sample, a total of 3.0 Gb sequence data was obtained.

### Whole cp genome and nrDNA assembly and annotation

Whole cp genomes of the samples were assembled using GetOrganelle v1.7.4 software (Jin et al., 2020). The published cp genome of *B. chinense* DC. (GenBank accession number: MN337347) was used as the reference genome (Zhang et al., 2019). Bowtie2 (Langmead and Salzberg, 2012) was first called to filter out plastid-like reads, and then SPAdes (Bankevich et al., 2012) was called for assembly. The cp genome was annotated using GeSeq (Tillich et al., 2017) and Plastid Genome Annotator (PGA) software (Qu et al., 2019), and the tRNAs in the genome were identified by tRNAscan-SE v2.0.7 (Lowe and Chan, 2016) and ARAGORN v1.2.38 (Laslett and Canbäck, 2004). The annotated genomes, together with the reference genome, were input into Geneious v22.2.2 (Kearse et al., 2012) for comparison to determine start and stop codons, as well as to manually correct the possible errors. Finally, the cp genome was physically mapped into a loop using the online software OGDRAW (Lohse et al., 2007). The assembled and annotated cp genome sequences were deposited in GenBank (http://www.ncbi.nlm.nih.gov/) under accession numbers OQ460227-OQ460239 (**Table 1**).

nrDNAs were also assembled using GerOrganelle v.1.7.3 software, and the output contigs were compared and annotated with the reference genome in Geneious v22.2.2.

### Genome structure and comparative analysis

The cp genome characteristics of the six species of *Bupleurum* were described, including the overall genome structure, the size of each part of the genome, and the GC content of the genes, which was acquired using Geneious v22.2.2. Multiple genome alignments of 13 sequences were done using the Mauve v2.4.0 plugin (Darling, 2004) in Geneious v22.2.2, with default parameters.

To determine the expansion/contraction of IR, genes distributed within and next to the boundaries of LSC, SSC and IR regions were compared and manually mapped to visualize them.

To investigate the highly variable regions (divergence hotspots), 13 complete chloroplast sequences of six *Bupleurum* species, were compared using MAFFTv. 1.3.7 (Katoh and Standley, 2013). Subsequently, the cp genomes of *Bupleurum* species were paired and visualized using the Shuffle LAGAN mode in mVISTA (Frazer et al., 2004), with the cp genome of *B. chinense* (GenBank access number: MN337347) as a reference. Using DnaSP v6.0 (Rozas et al., 2017), the nucleotide diversity (pi) in the cp genome sequence was calculated through sliding window analysis, with a window size of 600 bp and a step size of 200 bp.

Some other parameters, such as simple sequence repeats (SSRs), short dispersed repeats (SDRs), nonsynonymous (Ka) and synonymous (Ks) substitution rates, were also investigated. (**Supplementary File S1**, **Figures S1**, **2**).

### Molecular phylogenetic analysis

In order to get insight into the phylogenetic relationship among the *Bupleurum* species, maximum likelihood (ML) analyses and Bayesian inference (BI) analyses based on the whole cp genome, the coding sequences (CDS), the highly variable regions, the nrDNA, and the ITS region were performed respectively. Besides the sequences obtained in this study, the previously reported cp genome and ITS sequences of Chinese *Bupleurum* species were also downloaded from Genbank and incorporated into phylogenetic analysis (**Table 1**).

The best model was obtained under AIC criteria using ModelFinder (Kalyaanamoorthy et al., 2017) for molecular modeling of the dataset (**Table 2**). For ML analyses, IQ-tree 1.6.12 (Nguyen et al., 2015) was used, and the reliability of each branch was assessed using bootstrap values (number of replicates set to 1000). As for BI analyses, MrBayes 3.2.7a (Ronquist et al., 2012) was used, and Markov chain Monte Carlo (MCMC) analysis was run for a total of 20 million generations, with samples taken every 1000 generations. In the phylogenetic tree construction, *Centella asiatica* were used as outgroup species, and the resulting tree files were visualized and embellished using iTOL v.6.1.2 software (Letunic and Bork, 2021).

**Table 2:** Best models for ML and BI analyses.

dataset	whole chloroplast genome	the coding sequences (CDS)	nrDNA	ITS
ML	TVM+R10+F	GTR+F+R3	GTR+F+R5	GTR+F+G4
BI	GTR+F+I+G4	GTR+F+I+G4	GTR+F+G4	GTR+F+G4

## Results

### Chloroplast genome and nrDNA features

**Figure 1:**
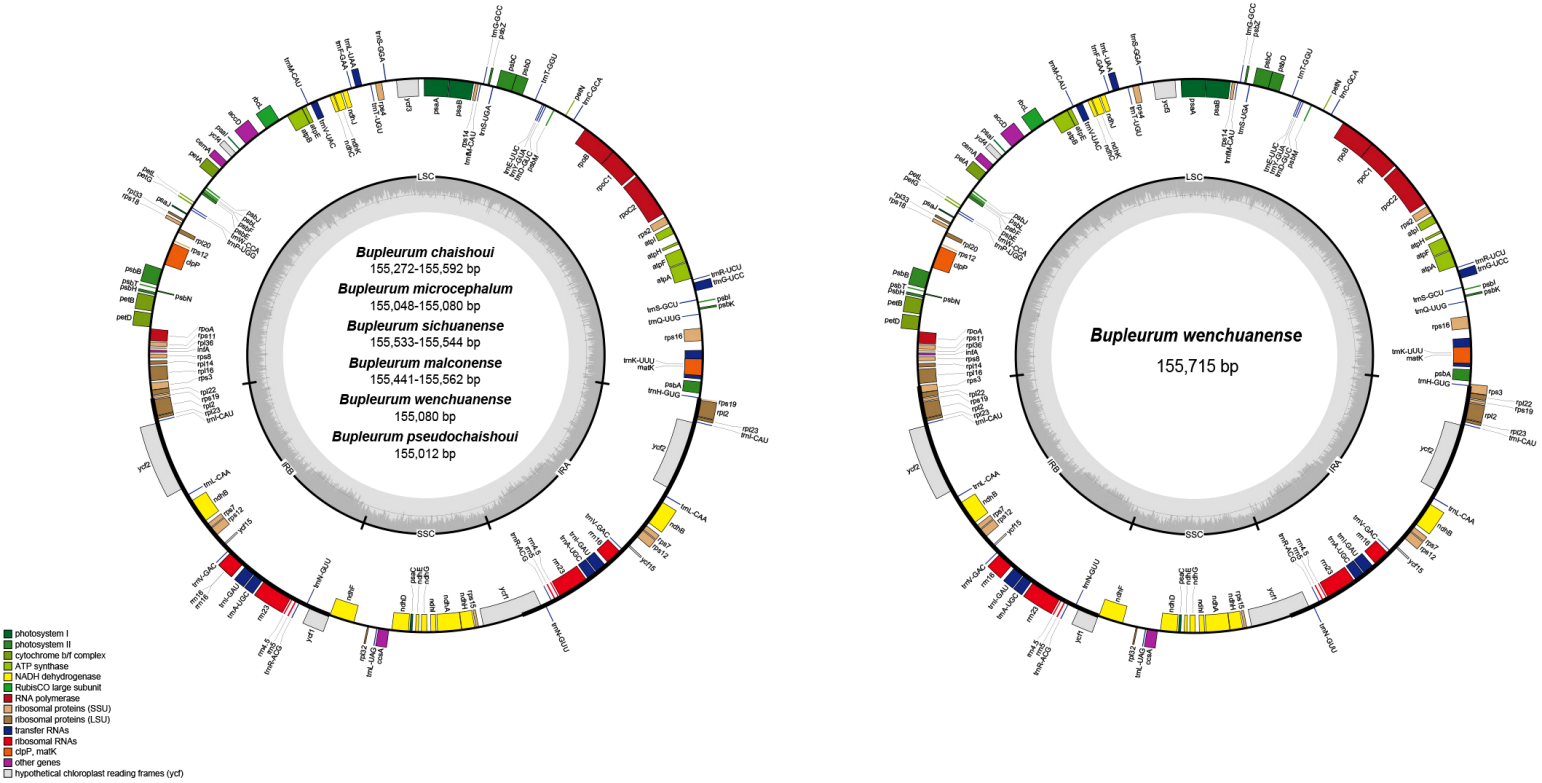
cp genome map of the six *Bupleurum* species Genes shown outside of the larger circle are transcribed clockwise, while genes shown inside are transcribed counterclockwise. Thick lines of the smaller circle indicate IRs and the inner circle represents the GC variation across the genic regions.

A total of 13 cp genomes from the six *Bupleurum* species were obtained. Size of these cp genomes ranges from 155,012 bp (*B. pseudochaishoui*) to 155,715 bp (*B. wenchuanense*). All of them exhibit a typical quadripartite structure, consisting of a small single copy (SSC) region (17,368-17,524 bp), a pair of inverted repeat (IR) regions (52,250-54,902 bp) and a large single copy (LSC) region (83,282-85,491 bp) (**Table 3**, **Figure 1**). The overall GC contents are highly similar (37.7-37.8%) (**Table 3**).

**Table 3:** Summary on chloroplast features of six *Bupleurum* species.

	*B. chaishoui*	*B. microcephalum*	*B. sichuanense*	*B. malconense*	*B. wenchuanense*	*B. pseudochaishoui*
Total length (bp)	155272-155592	155048-155080	155533-155544	155441-155562	155080-155715	155012
Total GC content (%)	37.7	37.8	37.7	37.7	37.8	37.8
IRa length (bp)	26295-26322	26125-26133	26295-26311	26309-26311	26129-27451	26134
IRb length (bp)	26295-26322	26125-26133	26295-26311	26309-26311	26129-27451	26134
SSC length (bp)	17368-17456	17511-17524	17493-17494	17468-17494	17521-17531	17530
LSC length (bp)	85314-85492	85258-85319	85428-85450	85325-85476	83282-85301	85214
Number unique genes	114	114	114	114	114	114
Protein coding	80	80	80	80	80	80
tRNA genes	30	30	30	30	30	30
rRNA genes	4	4	4	4	4	4

Genes and its arrangement in the cp genomes of the six *Bupleurum* species are almost the same. Each cp genome encodes 114 genes, including 80 protein-coding genes (PCG), 4 rRNA genes, and 30 tRNA genes. There’re 60 PCGs and 22 tRNA genes in the LSC region, 12 PCGs and 1 tRNA gene in the SSC region. In the IR region, for 12 of the 13 cp genomes, there’re 20 gene repeats, including 8 PCGs, 7 tRNA genes and 4 rRNA genes, except for one *B. wenchuanense* sample (voucher 2020080639), which has 2 extra PCGs (*rpl*22 and *rps*3) and thus a total of 22 genes in the IR region. Among these 114 genes, eighteen genes are found with introns, 15 of which containing one intron, while the other three (*clp*P, *ycf*3, and *rps*12) containing two introns. Incomplete copies of *ycf*1 and *rps*19 in the IR region are considered as pseudogenes. (**Table 3**, **Table 4**, **Figure 1**).

**Table 4:** List of genes encoded in six *Bupleurum* plastomes.

Gene Category	Genes	Number
Ribosomal RNAs	*rrn*4.5 ^(×2)^; *rrn*5 ^(×2)^; *rrn*16 ^(×2)^; *rrn*23 ^(×2)^	8
Transfer RNAs	t*rn*A-UGC ^(×2)a^; *trn*C-GCA; *trn*D-GUC; *trn*E-UUC; *trn*F-GAA; *trn*fM-CAU; *trn*G-GCC; *trn*G-UCC ^a^; *trn*H-GUG; *trn*I-CAU ^(×2)^; *trn*I-GAU ^(×2) a^; *trn*K-UUU ^a^; *trn*L-CAA^(×2)^; *trn*L-UAA ^a^; *trn*L-UAG; *trn*M-CAU; *trn*N-GUU ^(×2)^; *trn*P-UGG; *trn*Q-UUG; *trn*R-ACG ^(×2)^; *trn*R-UCU; *trn*S-GCU; *trn*S-GGA; *trn*S-UGA; *trn*T-GGU; *trn*T-UGU; *trn*V-GAC ^(×2)^; *trn*V-UAC ^a^; *trn*W-CCA; *trn*Y-GUA	37
Subunits of photosystem I	*psa*A; *psa*B; *psa*C; *psa*I; *psa*J; *ycf*3^b^; *ycf*4	7
Subunits of photosystem II	*psb*A; *psb*B; *psb*C; *psb*D; *psb*E; *psb*F; *psb*H; *psb*I; *psb*J; *psb*K; *psb*L; *psb*M; *psb*N; *psb*T; *psb*Z;	15
ATP-dependent protease subunit P	*clp*P^b^	1
Large subunit of rubisco	*rbc*L	1
NADH dehydrogenase	*ndh*A; *ndh*B ^(×2) a^; *ndh*C; *ndh*D; *ndh*E; *ndh*F; *ndh*G; *ndh*H; *ndh*I; *ndh*J; *ndh*K	12
Ribosomal protein (large subunit)	*rpl*2 ^(×2) a^; *rpl*14; *rpl*16 ^a^; *rpl*20; *rpl*22; *rpl*23 ^(×2)^; *rpl*33; *rpl*32; *rpl*36	11
Small subunit of ribosomal proteins	*rps*2; *rps*3; *rps*4; *rps*7 ^(×2)^; *rps*8; *rps*11; *rps*12 ^(×2)b^; *rps*14; *rps*15; *rps*16 ^a^; *rps*18; *rps*19	14
DNA-dependent RNA polymerase	*rpo*A; *rpo*B; *rpo*C1; *rpo*C2	4
Subunits of ATP synthase	*atp*A; *atp*B; *atp*E; *atp*F ^a^; *atp*H; *atp*I	6
C-type cytochrome synthesis gene	*ccs*A	1
Subunits of cytochrome b/f complex	*pet*N; *pet*A; *pet*L; *pet*G; *pet*B ^a^; *pet*D ^a^	6
Envelop membrane protein	*cem*A	1
Maturase	*mat*K	1
Hypothetical chloroplast reading frames	*ycf*1; *ycf*2 ^(×2)^	3
Subunits of Acetyl-CoA-carboxylase	*acc*D	1
Pseudogenes	*inf*A; *rps*19*; *ycf*1*; *ycf*15 ^(×2)^	5
Total	114 single-copy genes, 134 in total.	

(×2): Two gene copies in the IRs; a: Gene containing one intron; b: Gene containing two introns; * means the incomplete copy located in the IR of the gene straddling the IR and LSC/SSC regions.

Gene rps3 ^(×2)^and rpl22 ^(×2)^ are in one of the Bupleurum wenchuanense sequences.

The nrDNA sequences of the six *Bupleurum* species are 5821-5824 bp in length. The nrDNA is composed of five segments, i.e., the small-subunit rRNA gene (18s rDNA), internal transcribed spacer 1 (ITS1), the 5.8S rRNA gene, internal transcribed spacer 2 (ITS2), and the large-subunit rRNA gene (26s rDNA). The length of each segment is 1809 bp, 214-217 bp, 163-164 bp, 227-228 bp, and 3406-3407 bp respectively. The overall nrDNA GC content is 54.4%; among the five segments, ITS regions have higher GC content (with a value of 57.9-58.7%). (**Table 5**).

**Table 5:** Summary on nrDNA(18S-ITS1-5.8S-ITS2-28S) features of six *Bupleurum* species.

	*B. chaishoui*	*B. microcephalum*	*B. sichuanense*	*B. malconense*	*B. wenchuanense*	*B. pseudochaishoui*
Total length (bp)	5822	5823	5821	5821	5824	5824
18S rRNA length (bp)	1809	1809	1809	1809	1809	1809
ITS1 length (bp)	216	216-217	213-214	214-215	216	216
5.8S rRNA length (bp)	163	163-164	163-164	163-164	164	164
ITS2 length (bp)	227-228	227	228	227	228	228
26S rRNA length (bp)	3406-3407	3407	3407	3407	3407	3407
Total GC content (%)	54.4	54.4	54.4	54.4	54.4	54.4
ITS GC content (%)	58.3	58.0-58.2	58.7	58.8	57.9-58.4	58.4

### Comparative analysis of chloroplast genome and nrDNA structure

**Figure 2:**
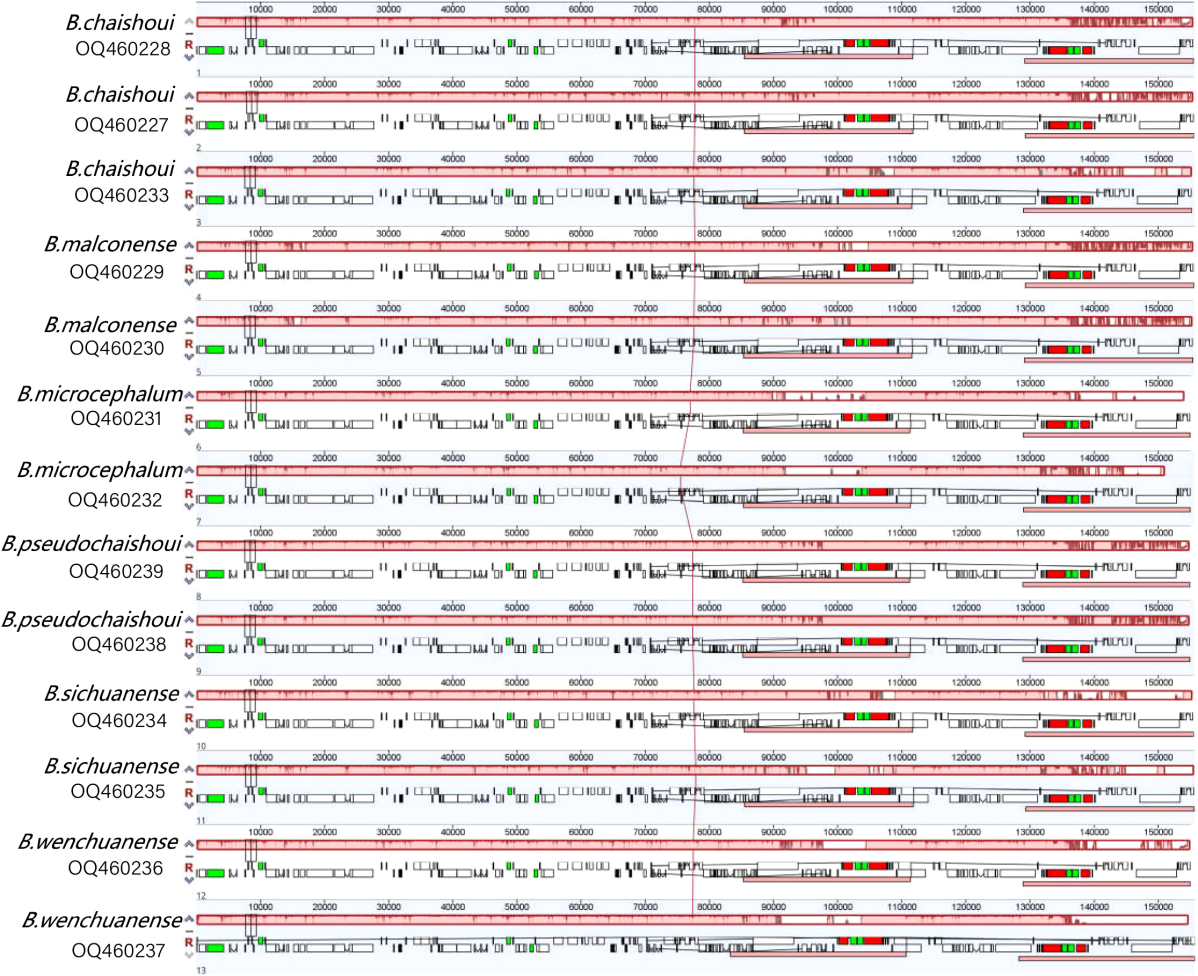
MAUVE alignment of the six *Bupleurum* species Locally collinear blocks are represented by continuously colored regions.

Results of Mauve pairwise analysis indicate that no rearrangement has occurred in coding and noncoding regions of the cp genomes of the six *Bupleurum* species (**Figure 2**), implying that the cp genomes are conservative.

**Figure 3:**
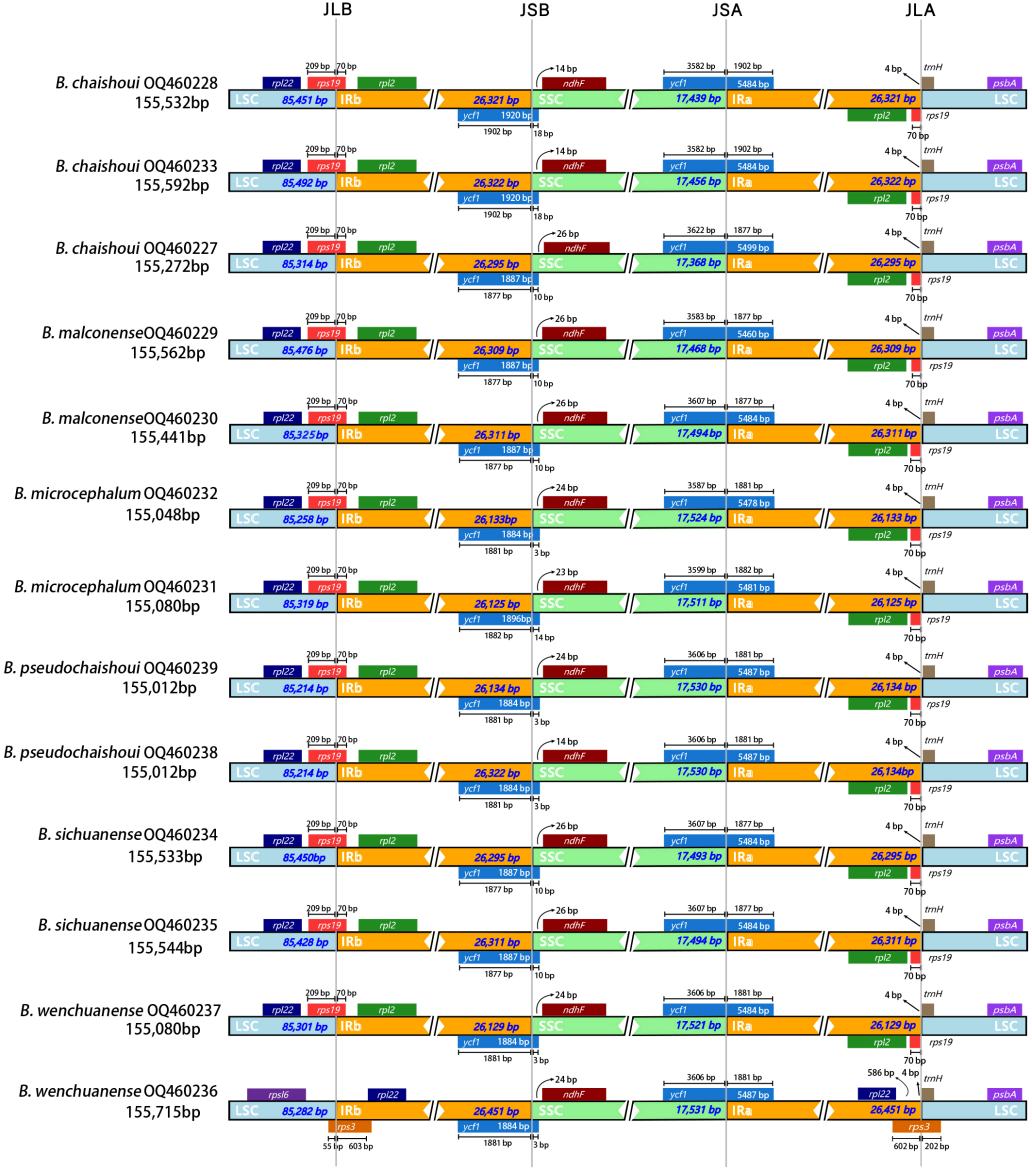
Comparison of the LSC, SSC and IR junction among the six *Bupleurum* species JLB, junction line between LSC and IRb; JSB, junction line between SSC and IRb; JSA, junction line between SSC and Ira; JLA, junction line between LSC and IRa.

The genes *rpl*22 and *rpl*2 are located on the left and right sides of the LSC/IRb junction respectively, while the gene *rps*19 crosses the JLB line (70 bp occurring in the IR region). The *ndh*F gene is at the right side of the IRb/SSC junction and 14-26 bp away from the boundary. Traversing the SSC and IRa region is the gene *ycf*1; a fragment of 1877-1902 bp in length of the gene is located in the IR region. The gene *trn*H lies 4-bp away from the JLA line (IRa/LSC boundary) to the right (**Figure 3**).

There’s an obvious expansion in the IR region of a *B. wenchuanense* sample (voucher 2020080639). In this sample, as mentioned above, genes *rpl*22 and *rps*3 are found in the IR region. Generally, these two genes were located in the LSC region. The *rps*3 gene is crossing the junction of LSC/IRb (JLB) and LSC/IRa (JLA). (**Figure 3**).

### Highly variable regions & nucleotide polymorphism in the chloroplast genomes and nrDNA

**Figure 4:**
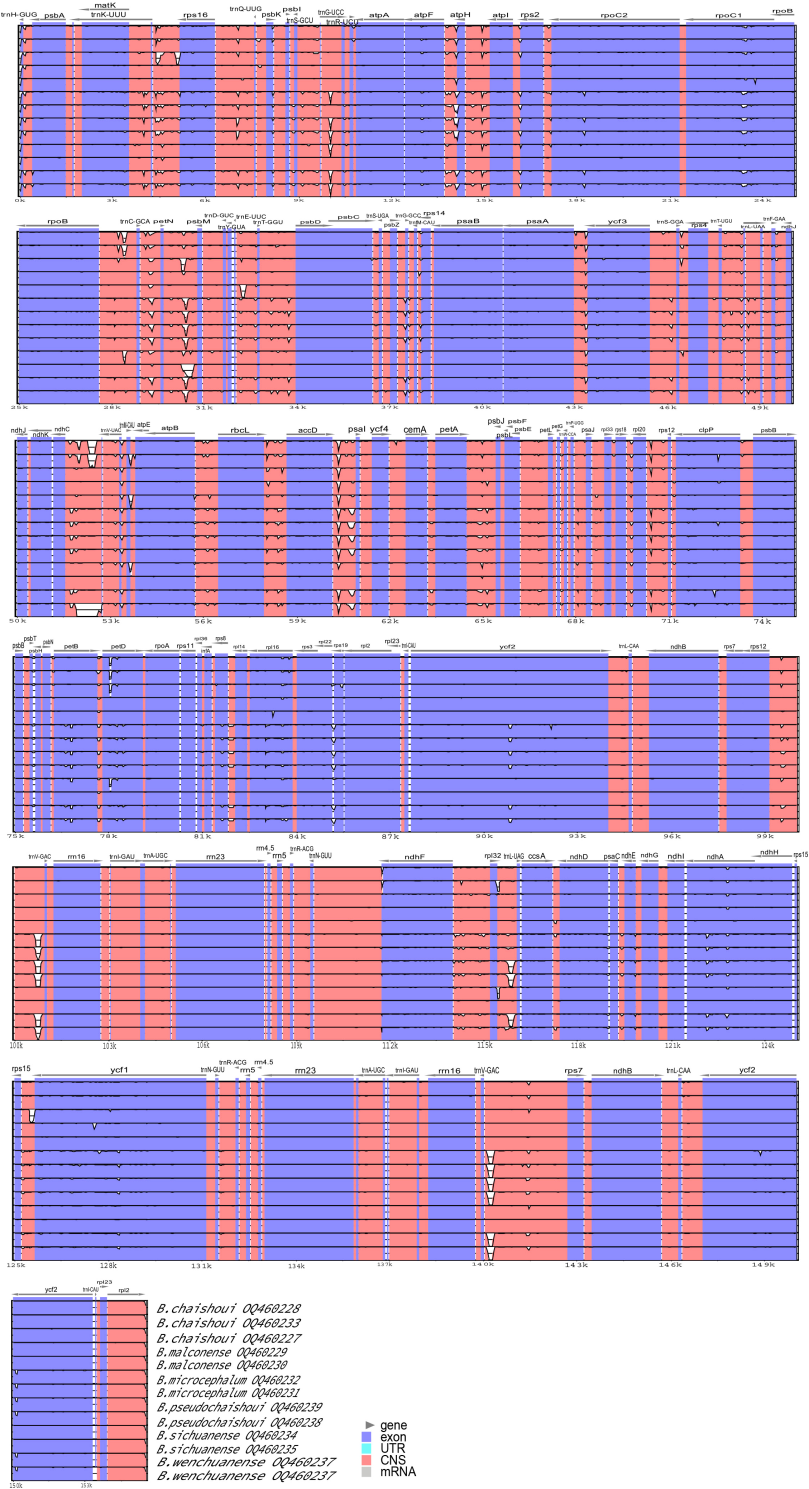
Comparison of the six *Bupleurum* species cp genomes using the mVISTA alignment program, with *B*. *chinense* as a reference.

We compared and mapped whole chloroplast gene alignments among 13 sequences of six *Bupleurum* species using mVISTA, using the published cp genome of *B. chinense* (No. MN337347) as the reference. The results showed that the divergence in the noncoding regions was greater than that in the coding regions, and a high degree of divergence in the 13 cp genomes appeared at intergenic spacers, including *trn*K-*rps*16, *atp*F-*atp*H, *atp*H-*atp*I, *trn*C-*pet*N, *pet*N-*pet*M, *ndh*C-*trn*V, *pet*A-*psb*J, *acc*D-*psa*I, *rps*15-*ycf*1, *rpl*32-*trn*L, and *trn*V-*rps*7. The coding regions were more conserved, and *trn*L, *rpo*C, *pet*D, *ycf*2, *ndh*A and *ycf*1 were the more divergent coding regions of these species. (**Figure 4**).

In order to assess the sequence divergence level of these *Bupleurum* species, the nucleotide diversity (Pi) of the cp genomes and nrDNAs were calculated.

**Figure 5:**
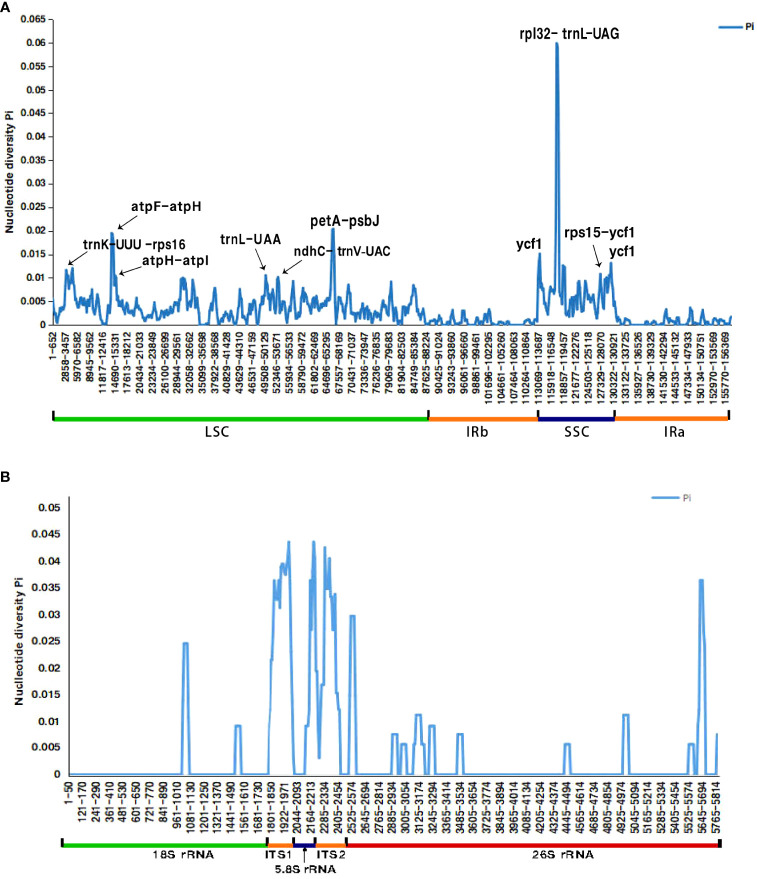
Comparative analysis of the nucleotide diversity (Pi) value among the six *Bupleurum* species **(A)** cp genome; **(B)** nrDNA X-axis: nucleotide position, Y-axis: nucleotide diversity value.

The Pi values of the whole cp genome of the six species range from 0.00-0.06, and the two IR regions were significantly less divergent than the LSC and SSC regions. The spacer regions between genes were found with higher Pi values than the coding regions. Totally there’re 7 spacer regions with Pi values greater than 0.01; the spacer with the highest Pi value was *rpl*32-*trn*L (Pi> 0.06), followed by *pet*A-*psb*J (Pi> 0.02), and consecutively, *trn*K-*rps*16, *atp*F-*atp*H, *atp*H-*atp*I, *ndh*C-*trn*V and *rps*15-*ycf*1 (Pi> 0.01). Only two coding regions (*trn*L-UAA and *ycf*1) had Pi values higher than 0.01, and the *ycf*1 gene has higher value. (**Figure 5A**).

The concatenated nrDNA sequences of the six *Bupleurum* species have 65 variation sites and 54 parsimony-informative sites, accounting for 1.12% and 0.93% of the total sequences, respectively. The Pi value of the whole sequence was 0-0.044, and the variant regions were mainly concentrated in the transcribed spacer region (ITS), with a mean value of 0.029 (range 0-0.044) for ITS1, 0.007 (range 00-0.036) for 5.8S rRNA and 0.023 (range 0-0.044) for ITS2. 18S rRNA and 26S rRNA were more conserved, with a mean value of 0.001 (range 0-0.025) for 18S rRNA and 0.002 (range 0-0.036) for 26S rRNA. (**Figure 5B**).

### Phylogenetic analysis

**Figure 6:**
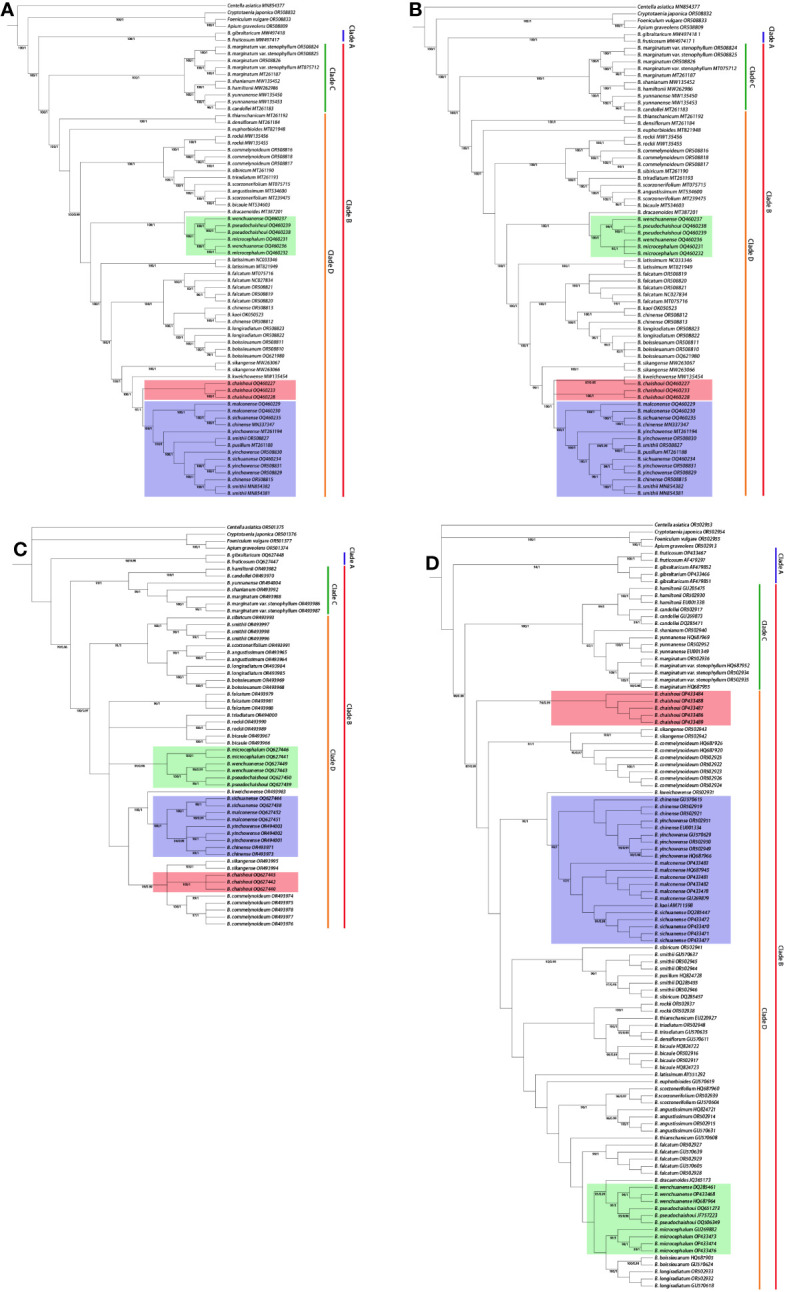
Phylogenetic trees showing the relationship between *Bupleurum* species. BS>70, PP>0.95 obtained by ML/BI methods are shown above the branches. **(A)** Phylogenetic tree based on the whole cp genome; **(B)** Phylogenetic tree based on protein-coding genes; **(C)** Phylogenetic tree based on the nrDNA; **(D)** Phylogenetic tree based on the ITS region.

**Figures 6A-D** shows the results of maximum likelihood (ML) analysis and Bayesian inference (BI) analysis of chloroplast whole genomes, protein-coding genes, the nrDNA and ITS region respectively, for the studied *Bupleurum* species. The phylogenetic trees estimated by ML and BI analyses showed similar topologies with high bootstrap support values and strong posterior probabilities for most nodes. The trees constructed based on the cp genome, protein-coding genes, and nrDNA were essentially alike. *Bupleurum* species formed a monophyletic clade which was sister to the other three species of Apioideae and could be divided into two main subclades, one consisting of *B. fruticosum* and *B. gibraltaricum* from the Mediterranean region (clade A), and the other consisting of all the remaining Chinese species (clade B).

The clade of Chinese species in subgenus *Bupleurum* can be further divided into clades C and D. In all trees, the constituent species of these two clades are consistent. A small one (clade C) was composed of *B. hamiltonii* N.P. Balakr., *B. candollei* Wall. ex DC., *B. yunnanense* Franch., *B. marginatum* Wall. ex DC. and its variety *B. marginatum* var. *stenophyllum* (H. Wolff) R.H. Shan & Y. Li. A large one (clade D) included more than 20 species; the six species produced in western Sichuan are all in this large clade.

Topology of clade D in the chloroplast-based trees and nuclear gene-based trees showed difference to some extent. The number of branches, the composing species of each branch, and the position of the branches, were quite different; for example, the position of *B. chaishoui*, the relationship between *B. microcephalum* and *B. wenchuanense* (See below, Discussion section, and **Figure 6**). Nevertheless, all four phylogenetic trees showed that the new species *B. pseudochaishoui* were closely related to *B. wenchuanense* and *B. microcephalum*; they were either forming/mixing in a separating branch (CP, CDS and nrDNA trees) or locating in adjacent branches (ITS tree). Similarly, *B. malconense* and *B. sichuanense* are closely related species of *B. chinense* and *B. yinchowense* R.H. Shan & Y. Li (**Figures 6A-D**).

The position of *B. chaishoui* varied in different trees. In the CP tree, it’s close to *B. malconense*-*B. sichuanense*-*B. chinense*-*B. yinchowense* group, and then merged with *B. kweichowense* R.H. Shan and *B. sikangense*. In the CDS tree, it joined with *B. kweichowense* first, then *B. malconense*-*B. sichuanense*-*B. chinense*-*B. yinchowense* group and *B. sikangense*. In the NR tree, it’s adjacent to the branch of *B. sikangense* and *B. commelynoideum* H. Boissieu, and then aggregated with *B. rockii* H. Wolff and *B. triradiatum* Adams ex Hoffm. successively. It’s noticeable that in the ITS tree, *B. chaishoui* formed an independent branch sister to other species, neighboring to *B. sikangense* and *B. commelynoideum* branch.

The nrDNA phylogenetic tree has the highest resolution in differentiating the six *Bupleurum* species, in which each species formed a monophyletic branch.

## Discussion

### Differences in gene and structure of chloroplast genomes among *Bupleurum* species

We described for the first time the cp genomes of six species of *Bupleurum* from western Sichuan Province, China, providing important insights into the cp genome characteristics of members of this genus. Our results show that the cp genomes of *Bupleurum* species are extremely similar, indicating that they are highly homogeneous in terms of cp genome structure, gene content and tendency of SDRs and SSRs. The cp genomes of the six *Bupleurum* species differ in size by only a few hundred bp (155,012-155,712 bp), consistent with the description that the cp genomes of *Bupleurum* species show only minor differences in size (~1 kb) (Huang et al., 2021a). Many mutational events occurring in the cp genome, including substitutions, insertions and deletions (InDels), inversions, genomic rearrangements and translocations (Abdullah et al., 2021), did not occur in any of the six *Bupleurum* species.

It has been reported that IR region contraction and expansion is a common phenomenon in cp genomes, and that changes in the size of the cp genome are usually the result of IR region expansion and that these variants can be observed in both closely and distantly related plant species and may lead to pseudogene production, gene duplication and deletion of individual copies of genes (Plunkett and Downie, 2000; Wang et al., 2008c; Saina et al., 2018). Comparative analysis of IR boundaries showed the same distribution of genes at the LSC/IR junction in the cp genomes of the six *Bupleurum* species, with minor differences in gene length (*ycf*1 and *rps*19) at the SSC/IR junction. The *ycf*1 sequence located at the IRa and SSC boundaries was identified as a pseudogene because it was truncated and was an incomplete duplicate of a normal copy. In the cp genome of a sample (*B. wenchuanense* voucher 2020080639), expansion of the IR region was found occurred, resulting in inconsistency between LSC/IR and SSC/IR border genes to other *Bupleurum*. The expansion was due to duplication of genes in IR (*rpl*22 and *rps*3). Variation in the number of duplicated genes in angiosperm cp genomes is common and different numbers of duplicated genes can be observed in different species (Ahmed et al., 2012; Menezes et al., 2018; Abdullah et al., 2020), but IR expansion within species is somewhat uncommon. This phenomenon was once observed in the cp genome of *Sinopodophyllum hexandrum* (Ma et al., 2022). We performed read mapping analysis and confirmed such an expansion in the very sample of *B. wenchuanense* (**Figure S4**). However, the overall gene number and order remains consistent and reflects the significant conserved nature of chloroplasts in this species.

### Highly variable regions in cp genome presumed as potential molecular markers

Inspired by Dong’s research work (Dong et al., 2012), highly variable regions in cp genome have been generally thought to be potential molecular markers for phylogeny and identification of plant species. In some cases, the highly variable regions worked well in resolving phylogeny and identifying species (Dong et al., 2021; Song et al., 2022), but in some other cases they were not so effective (Wu et al., 2020); more often, their effectiveness has not been evaluated (Liu K et al., 2021; Liu X.J. et al., 2021; Wu et al., 2022). In order to assess whether the they can work in *Bupleurum* phylogenetic analysis and species identification, we checked the highly variable regions in cp genomes of the six *Bupleurum* species.

We found that the non-coding intergenic spacer regions have considerable diversity over the protein-coding regions, which was consistent with most previous studies (Daniell et al., 2016; Javaid et al., 2022). Seven spacer regions (*rpl*32-*trn*L, *pet*A-*psb*J, *atp*F-*atp*H, *trn*K-*rps*16, *atp*H-*atp*I, *ndh*C-*trn*V, and *rps*15-*ycf*1), and two coding regions (*trn*L-UAA and *ycf*1) were found to be highly variable (Pi > 0.01). However, the discovered highly variable regions were not all the same as the previously reported ones in *Bupleurum* (Li et al., 2020; Huang et al., 2021a; Huang et al., 2021b; Xie et al., 2021). The spacer regions *pet*A-*psb*J, *trn*K-*rps*16 and the gene *ycf*1 are the highly variable regions found in common in these studies on *Bupleurum* cp genomes. There is evidence supporting the *ycf*1 gene as one of the core plastid DNA barcodes in land plants (Neubig et al., 2009; Franck et al., 2012; Dong et al., 2015). The *rpl*32-*trn*L, *pet*A-*psb*J gene have also been reported to show very high nucleotide per site diversity in several genera (Dong et al., 2012; Tan et al., 2020; Wang et al., 2022; Niu et al., 2023), and indeed the Pi values of the *rpl*32-*trn*L spacer regions of these six *Bupleurum* species were also much higher than the other regions in the present study, so *rpl*32-*trn*L together with *pet*A-*psb*J, *trn*K-*rps*16, and *ycf*1 were considered as having potential to be used as high-resolution DNA barcodes to identify species of *Bupleurum*.

We constructed phylogenetic trees of the six *Bupleurum* species using the four screened potential molecular markers independently and in combination, and found the trees were in similar topology to the whole cp genome and CDS trees, i.e., the six species were divided into two clades. But in each tree, there’s species group that cannot be clearly separated (**Figures S3A-F**). The “potential” disappears in reality. Therefore, we propose that in similar studies in the future such presumed molecular markers should be verified.

### Phylogenetic relationship inferred from cpDNA and nrDNA

The backbone of phylogenetic trees constructed based on the complete cp genomes, protein-coding sequences, nrDNA of the six *Bupleurum* species were highly silimar; and they were consistent with those acquired in the previous studies (Neves and Watson, 2004; Wang et al., 2008a; Wang et al., 2008b; Wang et al., 2011). The results confirmed *Bupleurum* is a monophyletic group and supported the treatment of its independent tribe status in subfamily Apioideae. The two main subclades represented the two subgenera in the genus, subgenus *Penninervia* and subgenus *Bupleurum*, respectively (Neves and Watson, 2004; Wang et al., 2011). The two clades (clade C and clade D in **Figure 6**) of Chinese *Bupleurum*, as well as the species included in each one, were also in concordance with the earlier researches (Wang et al., 2008b; Wang et al., 2011).

### B. microcephalum

In the six *Bupleurum* species in Western Sichuan, *B. microcephalum* and *B. malconense* were considered to be the most closely related species (Li and Sheh, 1979; She and Watson, 2005). The leaves of *B. microcephalum* are linear, extremely long, soft and thin, while the leaves of *B. malconense* are more than half shorter than that of *B. microcephalum*, and the texture is hard. But in all phylogenetic trees, *B. microcephalum* forms a clade with *B. wenchuanense*, and *B. pseudochaishoui*; in the tree based on nrDNA and ITS, it forms a sister branch to the latter two species. This is not consistent with the previous concepts that it’s close to *B. malconense*. The three species in this clade all have solitary stem, which is different from species that have caespitose rootstock and numerous stems in the other clade.

### B. sichuanense and B. malconense

*B. sichuanense* was established in 1992 by Pan and Hsu (Pan and Hsu, 1992). In the original species description, it was pointed out *B. sichuanense* was closely related to *B. malconense*, and was differ from the latter in its short and tender cauline leaves, more umbel rays (6~7), and bracteoles not longer than the pedicel (< 2 mm). In *Flora of China*, it was synonymized with *B. malconense* (She and Watson, 2005). Our molecular phylogenetic analysis proved their intimate relationships. In the phylogenetic trees constructed with the whole cp genomes, the CDS regions and the high variable regions, neither of the two formed a separate clade. In the nrDNA ITS tree, more than 10 sequences derived from these two species tangled together; but in the tree based on the nrDNA, *B. sichuanense* and *B. malconense* were clearly separated, implying they were two independent species. We checked the specimens of *B. malconense* and *B. sichuanense* deposited in Chinese Virtual Herbarium and those we collected and were of the opinion that the nrDNA correctly reflects the relationship between the two species. The results also implied the nrDNA might serve as a more suitable molecular marker for *Bupleurum* phylogenetic analysis and species identification.

The close relationship of *B. sichuanense* and *B. malconense* to *B. chinense* and *B. yinchowense* was disclosed almost 15 years ago (Xie et al., 2009). Their intimate relationship has once again been proven in this study. *B. chinense* is the officially recognized origin species of the crude drug of Chaihu (Chinese Pharmacopoeia Commission, 2020). There is a need for molecular marker to distinguish *B. chinense* from the other three closely related species (Chao et al., 2014). It seems nrDNA and ITS perform better than cpDNA does for such a purpose.

### B. chaishoui and the nucleocytoplasmic conflict

Phylogenetic trees constructed from cp genome and nrDNA show different topologies, indicating the existence of nuclear cytoplasmic conflicts. The position of *B. chaishoui* is an obvious example. Phylogenetic trees constructed from both nrDNA and ITS sequences support that *B. chaishoui* is closely related to *B. sikangense* and *B. commelynoideum*, while in the phylogenetic trees constructed from the whole cp genome sequence and CDS sequence, *B.chaishoui* is closely related to *B. malconense*; the latter is consistent with the opinion in FRPS that stated *B. chaishoui* and *B. malconense* are similar in morphology (Li and Sheh, 1979). The two species, together with the third species in the same clade, *B. sichuanense*, share a common morphological feature, i.e., the caespitose rootstock.

The inconsistencies in the topology of the gene trees acquired from the two different set of chloroplast and nuclear genomic data is a widespread phenomenon caused by a variety of reasons, including gene duplication and loss, horizontal gene transfer, incomplete lineage sorting, and hybridization introgression, etc. (Steenwyk et al., 2023). The most common reason for this phenomenon was believed to be chloroplast capture caused by hybridization (Fehrer et al., 2007; Galtier and Daubin, 2008; Soltis and Soltis, 2009; Stull et al., 2020). It was also reported cp genome-based phylogenetic relationships were often found to be related to geography rather than morphology (Liu et al., 2020). The Hengduan Mountains-Western Sichuan area is one of the diversity centers of *Bupleurum* species, and more than half of the species in China can be found in this region. The complex terrain and landforms, and diverse climate of ancient and modern times in the region have created opportunities for natural interspecific hybridization, and the results of phylogenetic trees suggest the possibility of ancient chloroplast capture events (Wang et al., 2008b; Sun et al., 2017; Yang et al., 2019; Xie et al., 2021). Moreover, the widespread presence of hybridization in biological taxa has gained increasing recognition (Fehrer et al, 2007; Acosta and Premoli, 2010). However, compared to its complex biodiversity, there are still very few clearly described hybridization events in the genus *Bupleurum*. In-depth researches in this aspect are expected in the future to reveal the important value of hybridization for the formation and maintenance of biodiversity of *Bupleurum*.

### The overlooked new species

The materials collected from Weizhou Town and Shuixi Town of Wenchuan (voucher Chao Zhi 1682502 and Chao Zhi & Huang Rong 2020080849) had been overlooked and mistakenly acknowledged as *B. chaishoui* for years, recently we realized it represents a new species and named it *B. pseudochaishoui*. These two samples formed distinct clade in all phylogenetic trees, supporting its status as an independent species.

**Figure 7:**
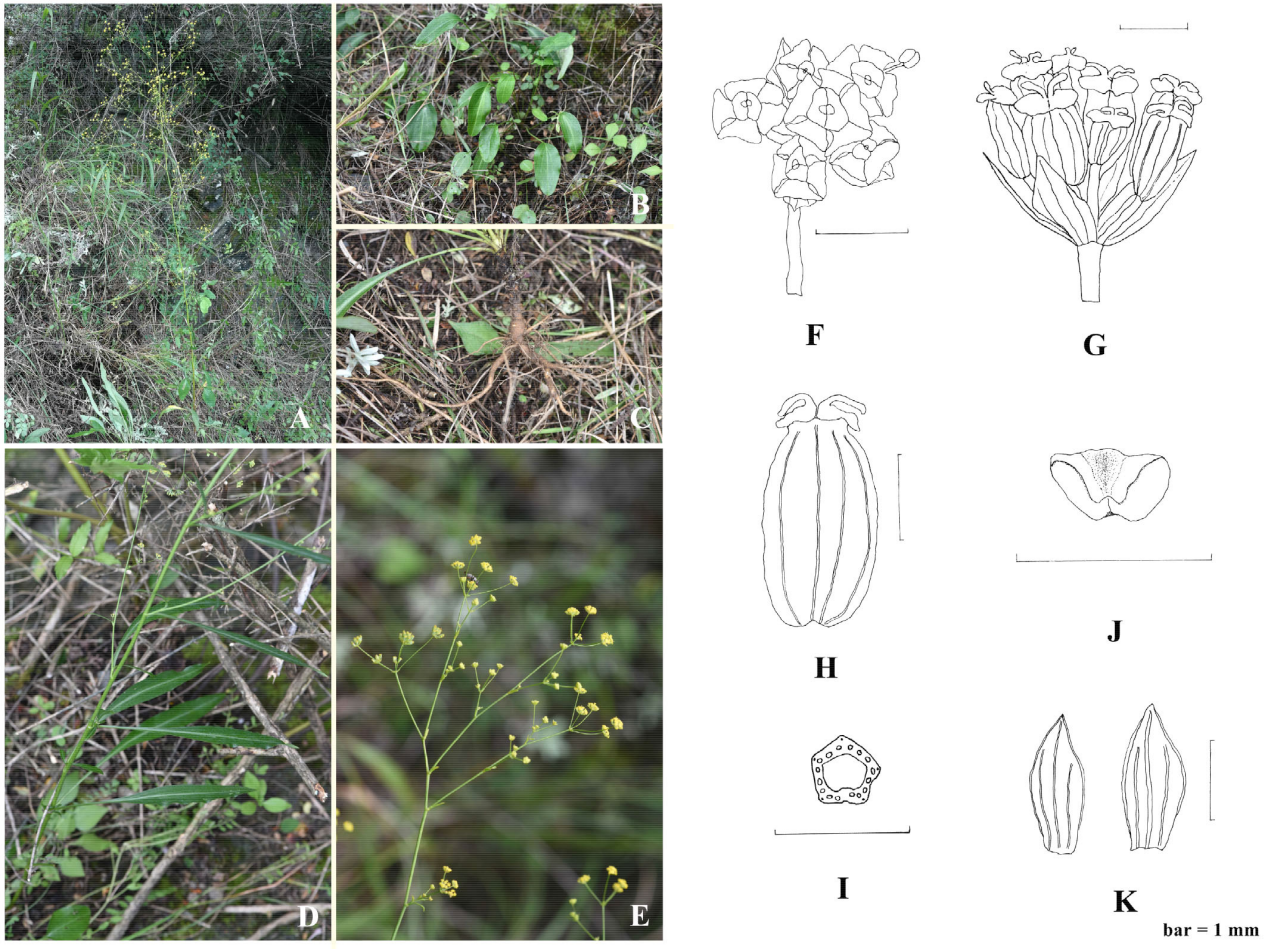
*Bupleurum pseudochaishoui* Z. Chao sp. nov. **(A)** habitat and individual plant; **(B)** basal leaves; **(C)** root; **(D)** middle cauline leaves; **(E)** Compound umbels; **(F)** umbellule; **(G)** infructescence; **(H)** cremocarp; **(J)** petal; **(K)** bracteole; **(I)** schizocarp transverse section.

*B. pseudochaishoui* shows distinguishable morphological character to its allies. Its leaves are coriaceous and some shiny. The basal leaves are long petiolated and the blades are quite large, blade broadly ovate-elliptic to lanceolate elliptic; the middle and upper cauline leaves are much smaller, long lanceolate, with no petiole (**Figure 7**). In Chinese *Bupleurum*, only *B. longiradiatum* Turcz. (Northeastern, Northern and Northwestern China) and *B. aureum* Fisch. ex Hoffm. (Xinjiang) have basal leaves with blades of such a similar shape and size to *B. pseudochaishoui*.

Molecular data indicated that *B. wenchuanense* is the most intimately related species to *B. pseudochaishoui*, but it differs from the new species in its numerous, rosette-caespitose oblanceolate basal leaves and subulate to squamose middle and upper leaves, and the (1–)2–3 very unequally rayed umbels, 1–4-flowered umbellules.

**Figure 8:**
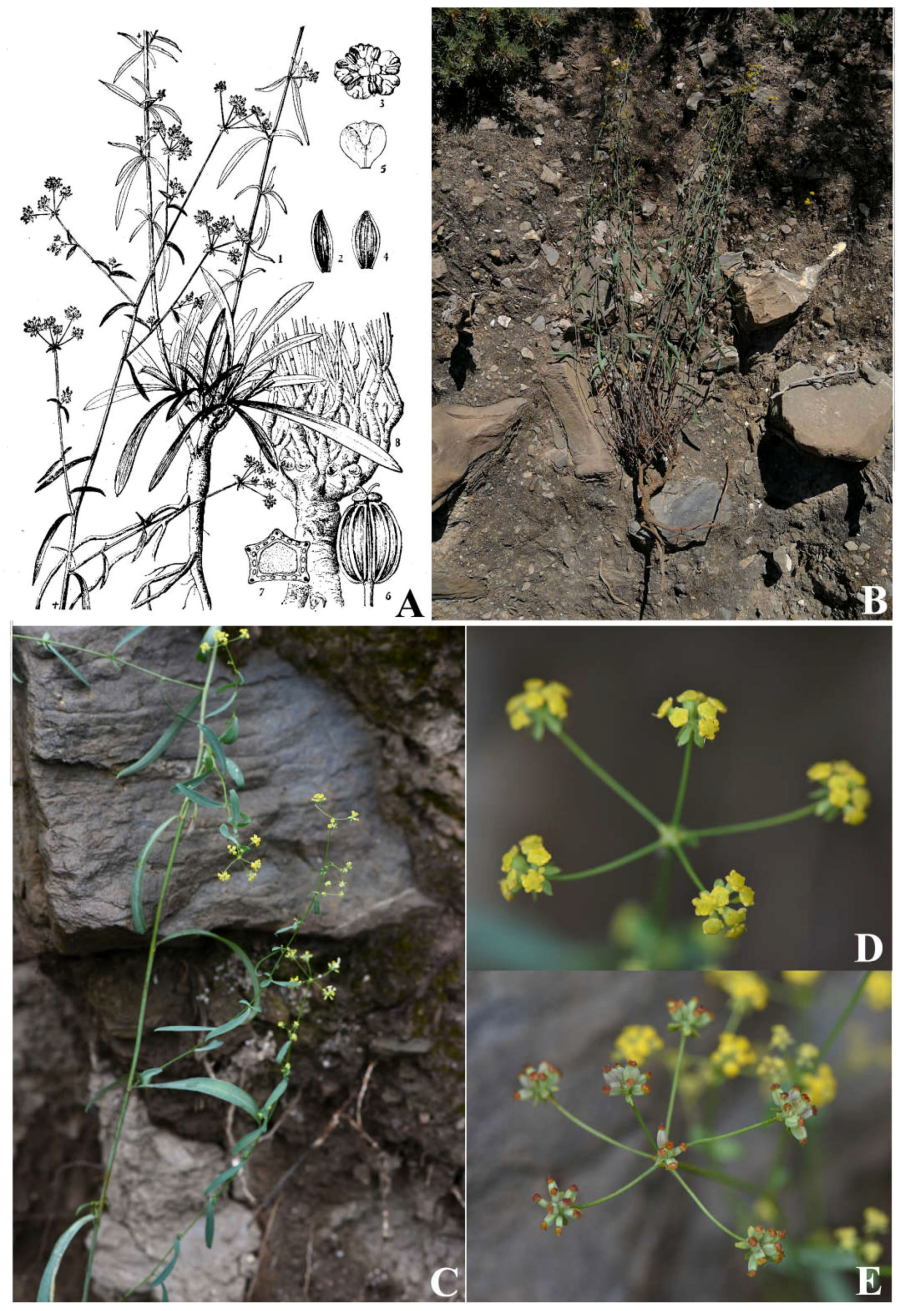
*Bupleurum chaishoui* R.H. Shan et M.L. Sheh **(A)** Illustration in FRPS; **(B)** individual plant; **(C)** middle cauline leaves; **(D)** Compound umbels; **(E)** infructescence.

*B. chaishoui*, the species which we previously misrecognized as *B. pseudochaishoui*, has caespitose numerous stems, and lanceolate to elliptic cauline leaves; the shape and size of cauline leaves vary greatly, especially those born on the same node or adjacent nodes are very unequal; the upper ones often grow in a reflexed way (**Figure 8**). On the contrary, *B. pseudochaishoui* has solitary stem, and its cauline leaves often are not reflexed.

In the areas neighbor to Southwestern China, there are another two *Bupleurum* species having basal leaves with broadly oval lamina, namely *B. lanceolatum* Wall. ex DC. in Himalayas in India and Western Pakistan and *B. gilesii* H. Wolff in Western Pakistan and Afghanistan (Missouri Botanical Garden, 2023). The upper cauline leaves are of similar shape to the basal and lower leaves in *B. lanceolatum*, and short condensed main stem and numerous branches from the base in *B. gilesii*, make them easily to be distinguished from *B. pseudochaishoui*.

## Conclusion

Comparative analysis of the cp genomes and nrDNA sequences of six *Bupleurum* species from western Sichuan Province, China revealed their conserved structure. Phylogenetic analysis based on nrDNA proved the monophyly of each species. Incongruence between nuclear and plastome gene trees was observed, which may be attributed to chloroplast capture. For overall consideration, *B. sichuanense* was thought to be an independent species different from *B. malconense*; *B. chaishoui* was supposed to be close relative to these two species, and they all shared the characteristic of having caespitose numerous stems. The other three species with solitary stem, *B. microcephalum*, *B. wenchuanense* and *B. pseudochaishoui*, were close in consanguinity. The new species, *B. pseudochaishoui*, was described and illustrated.

### Taxonomy

*Bupleurum pseudochaishoui* Z. Chao, sp. *nov.* (**Figure 7**).

Diagnosis:—This new species is easily distinguishable in the genus by its leathery dimorphic leaves, i.e., basal leaves with relatively large ovate-lanceolate blade and extremely long petiole, and lanceolate middle leaves. Molecular data implied it is closely related to *B. wenchuanense*, but the latter differs in its numerous, rosette-caespitose oblanceolate basal leaves and subulate to squamose middle and upper leaves, and the (1–)2–3 very unequally rayed umbels, 1–4-flowered umbellules.

Type:—CHINA. Sichuan Province: Wenchuan county, Buwa Village, growing on mountain slopes, 31°29′42.24″N, 103°35′43.01″E, elevation 2000 m, 25 August 2016 (fl. & fr.), *Chao Zhi 1682502* (holotype, Southern Medical University herbarium, SMU)!.

Description:—Perennial herb. Taproot 10-15 cm long and 5-8 mm in diameter, stout, brownish, the head often inflated and annulated, lower part always branched and longitudinally fine-wrinkled, with sparse small horizontal protuberance. Rhizome present at the top of the root, lignified, 3-10 cm long. Stem solitary, solid and rigid, surface obviously striped, 80-120 cm high; base woody, sometimes tinged purple; middle and upper part slightly tortuous, much branched, branches 10-30 cm long. Leaves leathery, bright green, abaxial surface light green, margin narrowly white-cartilaginous; basal and the lower leaves large, blade broadly ovate-elliptic to lanceolate elliptic, 5-13 cm × 2.5-4.5 cm, 5-7 nerved, apex rounded-obtuse or acute, base cuneate or broadly cuneate, tapering into long petiole up to 17 cm, slightly expanded into a sheath at the base, clasping; middle-part leaves long lanceolate, 10 cm × 1 cm, nerves 5-7, often white-colored on both sides of the midvein, apex acute or acuminate, base tapering and clasping; upper leaves gradually smaller. Inflorescence much-branched, umbels numerous, 1.5-3 cm across; bracts 1-5, ovate or squamose, unequal, 1-3 mm × 0.2-1 mm, 1-3 veined; rays 3-5, slender, unequal, 0.5-2.5 cm long; bracteoles 5, slightly membranous, ovate or lanceolate, 1.5-2 mm × 0.8-1 mm, longer than pedicels, slightly shorter than umbellules, apex acuminate, 3-veined; umbellules 2-4 mm across, (5) 8-10 (12)-flowered; florets about 1 mm in diameter. Petals pale yellow, slight brown convex at the top of the inflex, ligule slightly ladder-shaped, apex obviously 2-lobed, lobes obtuse; pedicels 1-1.5 mm long; stylopodium yellow, thick discoid, wider than ovary. Fruit oblong, 2 mm ×1 mm, brownish brown to dark brown, ribs not very obvious, vittae 3 in each furrow, 4 on commissure; fruit stalk about 2 mm long. Fl. Jul–Sep, fr. Aug–Oct.

Habitat and distribution:—The new species was found only in its type locality. It inhabits shrubs on mountain slopes, at an elevation about 2000 m. Fl. and fr. July-Sept.

Etymology:—The epithet refers to it has been previously mistaken as *B. chaishoui*.

A key to the six most closely related species of *Bupleurum* in the region of Western Sichuan is given as follows:

1. Stem usually numerous, caespitose.
2. Cauline leaves lanceolate to elliptic, very unequal at the same node, 1.2-9 cm × 0.3-1.2 cm, upper leaves usually reflexed ……………………*B. chaishoui* R.H. Shan & M.L. Sheh
2. Cauline leaves linear, similar in size, upper leaves not reflexed.
3. Involucel bracts less than 2 mm long, shorter than the pedicel; leaves tender, at most 8 cm long; rays of umbel usually 6-7 ………………………………… *B. sichuanense* S.L. Pan & P.S. Hsu
3. Involucel bracts more than 2 mm long, exceeding the pedicel; leaves firm, up to 15 cm long; rays of umbel usually less than 5………………………………………*B. malconense* R.H. Shan & Y. Li
1. Stem single, not caespitose.
4. Rays of umbel (1)2-3, very unequal; upper leaves small, subulate or squamose, few; stem much-branched ……………………*B. wenchuanense* R.H. Shan & Y. Li
4. Rays of umbel more than 4; leaves conspicuous and numerous.
5. Basal and cauline leaves uniform, linear, very long and narrow, herbaceous, soft, abaxially slightly glaucous ……………… *B. microcephalum* Diels.
5. Leaves coriaceous; the basal and lower ones large, blade broadly ovate-elliptic to lanceolate elliptic, petiole quite long; the middle and upper ones long lanceolate ………………… *B. pseudochaishoui* Z. Chao sp. nov.

## Data availability statement

The datasets presented in this study can be found in online repositories. The names of the repository/repositories and accession number(s) can be found below: https://www.ncbi.nlm.nih.gov/, OR501374-OR501377, OR502913-OR502955, OR508809-OR508833, OQ645436, OQ651273, OQ627438-OQ627452, OR493964-OR494004, OP433466-OP433489, OQ460227-OQ460239.

## Author contributions

ZC: Conceptualization, Funding acquisition, Resources, Investigation, Formal analysis, Writing – original draft, Writing – review & editing. XH: Methodology, Formal analysis, Visualization, Writing – original draft, Writing – review & editing. XX: Methodology, Formal analysis. RH: Methodology, Resources, Formal analysis. ET: Resources.

## Supplementary material

Supplementary Figure 1 Simple sequence repeats (SSRs) and short dispersed repeats (SDRs) in the six *Bupleurum* species.

Supplementary Figure 2 Selective pressure in six *Bupleurum* species.

Supplementary Figure 3 Phylogenetic trees constructed with highly variable regions.

Supplementary Figure 4 Read mapping of Bupleurum wenchuanense (voucher 2020080639 and 1683103) IR regions

Supplementary File 1 Simple sequence repeats (SSRs) and short dispersed repeats (SDRs) detection, and nonsynonymous (Ka) and synonymous (Ks) substitution rates analysis

Supplementary File 2 Key to Chinese Bupleurum (modified from Flora of China)
