# Supplementary figures and images for: Phylogenomic analysis of Bupleurum in Western Sichuan, China, including an overlooked new species
Source: Front Plant Sci. 2023 Nov 27;14:1294670. doi: 10.3389/fpls.2023.1294670 (PMC10715590; doi:10.3389/fpls.2023.1294670)

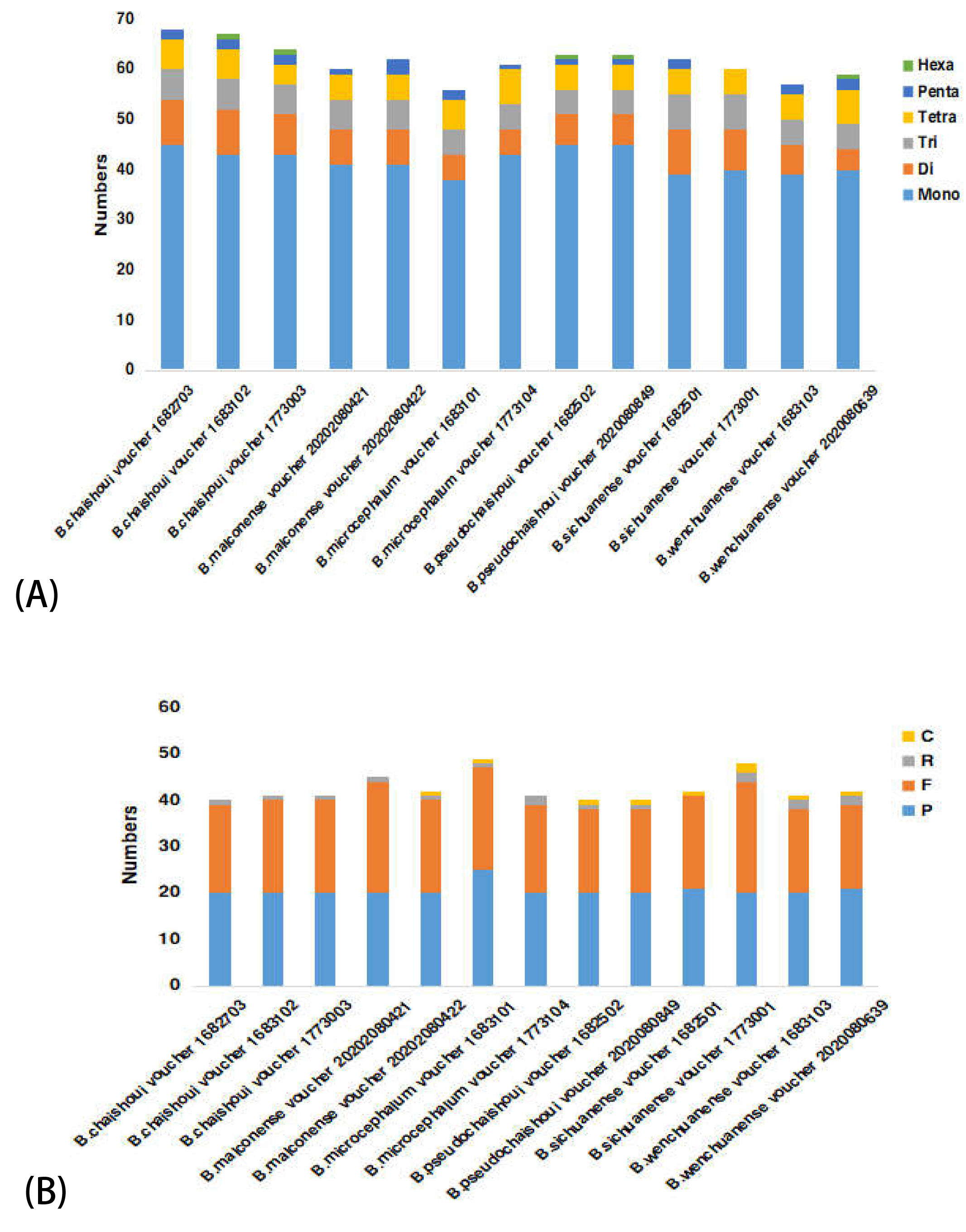

Supplement: Supplementary Figure 1 — Simple sequence repeats (SSRs) and short dispersed repeats (SDRs) in the six Bupleurum species. [file Image_1.jpeg]

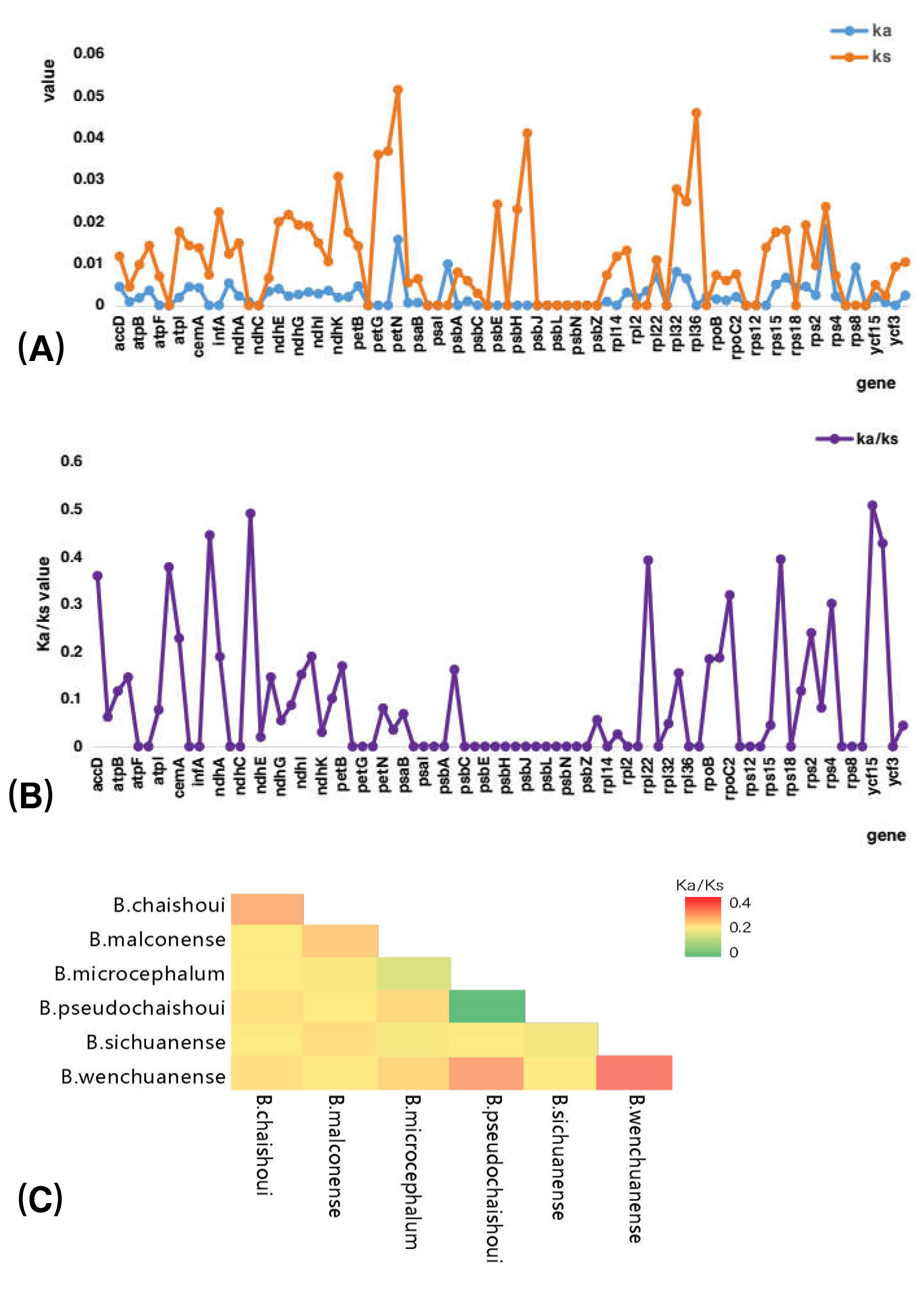

Supplement: Supplementary Figure 2 — Selective pressure in six Bupleurum species. [file Image_2.jpeg]

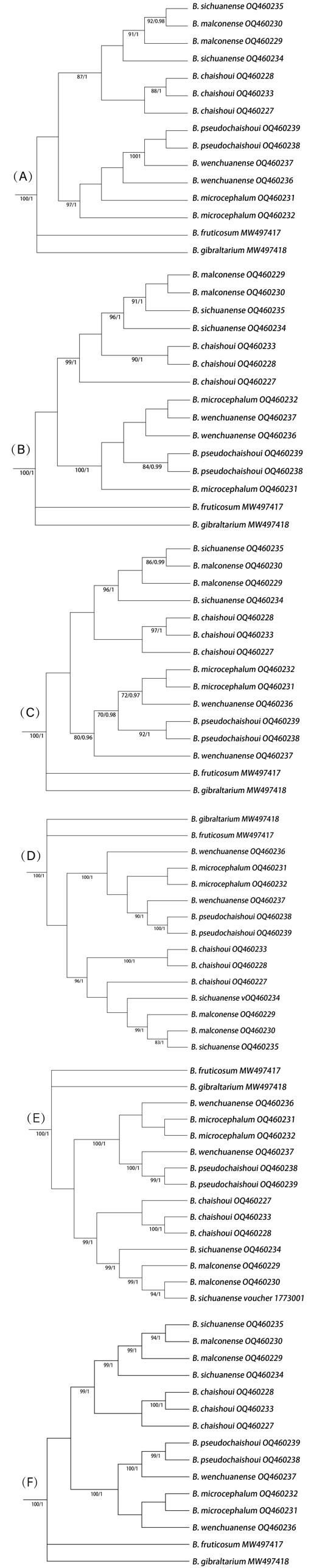

Supplement: Supplementary Figure 3 — Phylogenetic trees constructed with highly variable regions. [file Image_3.jpeg]

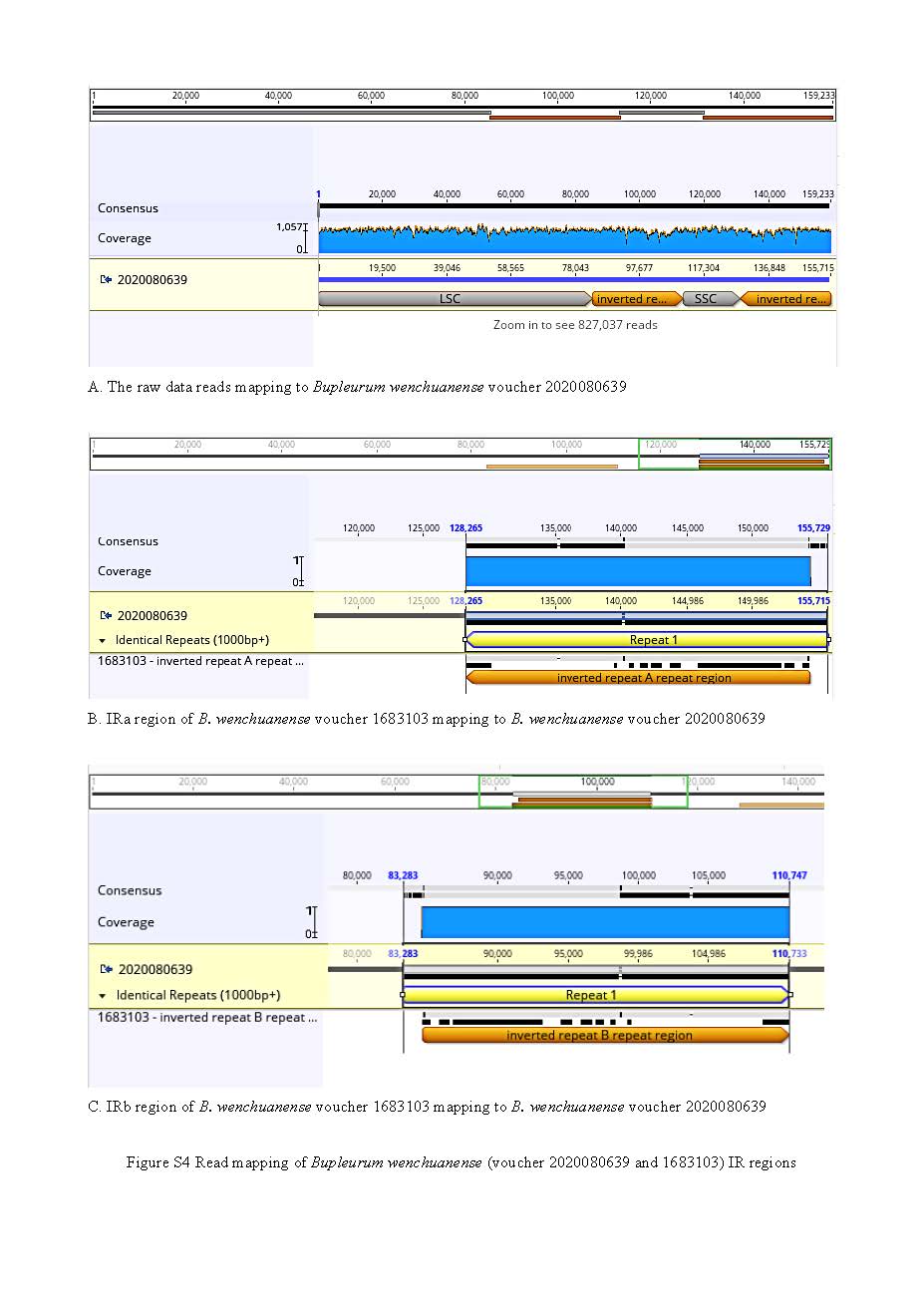

Supplement: Supplementary Figure 4 — Read mapping of Bupleurum wenchuanense (voucher 2020080639 and 1683103) IR regions [file Image_4.jpeg]
