# Supplementary material for: Phylogenomic analysis of Bupleurum in Western Sichuan, China, including an overlooked new species
Source: Front Plant Sci. 2023 Nov 27;14:1294670. doi: 10.3389/fpls.2023.1294670 (PMC10715590; doi:10.3389/fpls.2023.1294670)
Supplement: Supplementary File 1 — Simple sequence repeats (SSRs) and short dispersed repeats (SDRs) detection, and nonsynonymous (Ka) and synonymous (Ks) substitution rates analysis [file DataSheet_1.pdf]

Key to Chinese *Bupleurum* (modified from *Flora of China*)

1	Bracteoles large and conspicuous, mostly exceeding the umbellule.	(2)
+	Bracteoles small, narrow, usually shorter than, equaling or slightly exceeding the umbellules.	(16)
2(1)	Cauline leaves broadly ovate, 10–20 × 3–5.5 cm, base dilated, rounded, perfoliate (Xinjiang).	*Bupleurum aureum*
+	Cauline leaves linear, lanceolate or narrowly ovate, 1–15 × 0.1–1 cm, base dilated or not, but never perfoliate.	(3)
3(2)	Plants small, 7–20(–25 cm).	(4)
+	Plants tall, 25–125 cm.	(7)
4(3)	Bracteoles yellow or tinged purple.	(5)
+	Bracteoles green.	(6)
5(4)	Plants erect; bracteoles 5–8, yellow-green; stylopodium dark yellow (NW China).	*Bupleurum triradiatum*
+	Plants decumbent; bracteoles 6–10, tinged purple; stylopodium dark purple (NW and SW China).	*Bupleurum dalhousieanum*
6(4)	Leaves and bracteoles abaxially glaucous, 3–5-nerved; umbellules 10–20-flowered (Qinghai, Xinjiang).	*Bupleurum densiflorum*
+	Leaves and bracteoles abaxially not glaucous, 9–15-nerved; umbellules 8–14-flowered (Sichuan, Xizang, Yunnan).	*Bupleurum yunnanense*
7(3)	Middle and upper leaves base dilated, cordate and clasping.	(8)
+	Middle and upper leaves base not dilated nor cordate.	(9)
8(7)	Root dark brown; bracteoles usually 6–9; petals yellow-green (NC and NW China).	*Bupleurum smithii*
+	Root gray-brown; bracteoles usually 10–12; petals usually dark purple (C, NW, and SW China).	*Bupleurum longicaule*
9(7)	Inflorescence many-branched, forming a panicle (Yunnan).	*Bupleurum luxiense*
+	Inflorescence little-branched, not forming a panicle.	(10)
10(9)	Petals purple or dark purple (at least abaxially).	(11)

+	Petals yellow.	(12)
11(10)	Bracteoles 5(–7), green, broadly ovate, apex rounded or acute, apiculate (Jilin).	*Bupleurum euphorbioides*
+	Bracteoles 7–9, bluish-purple, elliptic, apex acuminate, long-apiculate (NW and SW China).	*Bupleurum commelynoideum*
12(10)	Lateral umbel present at base of terminal umbel and overtopping it (Guizhou).	*Bupleurum kweichowense*
+	Terminal umbel without a lateral umbel at base.	(13)
13(12)	Bracts 1–2, linear-lanceolate, usually deciduous (NC and NE China).	*Bupleurum sibiricum*
+	Bracts 1–5, elliptic or ovate, persistent.	(14)
14(13)	Bracts elliptic, apex acuminate; rays 8–11(NW and SW China).	*Bupleurum petiolulatum*
+	Bracts ovate or suborbicular, apex rounded or obtuse; rays 4–12.	(15)
15(14)	Leaves thin-papery, abaxially grayish-white-green, margin not thickened, green; rays 4–8 (Sichuan, Xizang, Yunnan).	*Bupleurum candollei*
+	Leaves thick-papery, often tinged reddish-brown, abaxially green, margins thickened and reddish; rays 7–12 (Sichuan, Yunnan).	*Bupleurum rockii*
16(1)	Shrub with woody stem 50–100 cm long	*Bupleurum dracaenoides*
+	Herbs	(17)
(17)	Leaves 8–25 × 2.5–10 cm, base dilated, cordate and clasping.	*Bupleurum longiradiatum*
+	Leaves 2–16(–20) × 0.1–1(–3) cm, base not dilated nor cordate and clasping.	(18)
18(17)	Plants small, 2–20(–30) cm (sometimes more in *B. gracillimum*).	(19)
+	Plants tall, (25–)30–125 cm (or less in *B. angustissimum* and *B. hamiltonii*).	(23)
19(18)	Stem base densely clothed with fibrous remnant sheaths.	(20)

+	Stem base without fibrous remnant sheaths (Qinghai, Sichuan, Xizang).	(21)
20(19)	Plants 15–30 cm; fruit 2.5–3 mm, ribs prominent; altitude 650–1550 m (NC and NE China).	*Bupleurum bicaule*
+	Plants 2–10 cm; fruit 3.5–4 mm, ribs conspicuous or narrowly winged; altitude 2300–3500 m (Nei Mongol, Ningxia, Qinghai, Xinjiang).	*Bupleurum pusillum*
21(19)	Plants gray-green; fruit ribs broadly winged (Xizang).	*Bupleurum alatum*
+	Plants tinged red; fruit ribs rounded or slightly prominent.	(22)
22(21)	Basal leaves very numerous, rosette-caespitose; rays 4–7, 3–6 cm; petals usually yellow or yellow-green (Qinghai).	*Bupleurum condensatum*
+	Basal leaves few, not rosette-caespitose; rays ca. 3, 0.5–1.7 cm; petals yellow or dark purple (Sichuan).	*Bupleurum gracillimum*
23(18)	Root surface reddish-brown.	(24)
+	Root surface usually gray-yellow or brown, not reddish-brown.	(26)
24(23)	Stem base without fibrous remnant sheaths (Gansu, Nei Mongol, Ningxia, Shaanxi).	*Bupleurum yinchowense*
+	Stem base clothed in fibrous remnant sheaths.	(25)
25(24)	Leaves linear, 6–16 × 2–7 mm (E, NC, NW, and SC China).	*Bupleurum scorzonerifolium*
+	Leaves narrow-linear, 6–18 × 0.8–1 mm (NC and NW China).	*Bupleurum angustissimum*
26(23)	Fruit vittae 1 in each furrow, 2 on commissure (C, SC, and SW China).	*Bupleurum hamiltonii*
+	Fruit vittae 1–3 in each furrow, 2–4 on commissure.	(27)
27(26)	Leaves lanceolate or oblong-elliptic, (0.5–)0.8–3 cm wide.	(28)
+	Leaves linear, 0.2–0.5(–7) cm wide (to 1 cm in *B. microcephalum*).	(34)
28(27)	Rootstock short, tuberous, with many fibrous fascicled roots (Heilongjiang, Jilin).	*Bupleurum komarovianum*

+	Taproot little-branched or unbranched.	(29)
29(28)	Leaf margin white cartilaginous (C, NW, and SW China).	*Bupleurum marginatum*
+	Leaf margin not white cartilaginous.	(30)
30(29)	Fruit vittae 1(rarely 2–3) in each furrow, 2 on commissure (Xinjiang).	*Bupleurum krylovianum*
+	Fruit vittae 3–4 in each furrow, 4 on commissure.	(31)
31(30)	Leaves coriaceous; the basal and lower ones large, blade broadly ovate-elliptic to lanceolate elliptic, petiole quite long; the middle and upper ones long lanceolate	*Bupleurum pseudochaishoui* sp. nov.
+	Leaves herbaceous; basal leaves and cauline leaves not obviously dimorphic	(32)
32(31)	Stem and branches usually slender and flexuose; bracts narrowly linear, 1–5 × 0.5–1 mm (C, E, NC, NE, and NW China).	*Bupleurum chinense*
+	Stem and branches rigid, not flexuous; bracts lanceolate, ovate, elliptic or obovate, 3–10 × 1–5 mm.	(33)
33(32)	Bracts 3–5, ovate, elliptic or obovate; fruit brown, glaucous (Chongqing).	*Bupleurum gracilipes*
	Bracts 2–3, lanceolate; fruit brown but not glaucous (Taiwan).	*Bupleurum kaoi*
34(27)	Stem solitary.	(35)
+	Stems numerous, often caespitose from a thickened woody caudex.	(39)
35(34)	Rays 1–3, filiform, very unequal, remote (NW Sichuan).	*Bupleurum wenchuanense*
+	Rays (3–)4–10, not slender, moderately equal.	(36)
36(35)	Fruit oblong; ribs prominent.	(37)
+	Stem little–branched; fruit ovoid or ellipsoid; ribs inconspicuous.	(38)
37(36)	Stem little-branched (W Xinjiang).	*Bupleurum thianschanicum*

+	Stem many-branched (Yunnan).	*Bupleurum polyclonum*
38(36)	Pedicels 1–1.5 mm; fruit ovoid; mericarp pentagonal in cross section (Gansu, Sichuan, Xizang)	*Bupleurum microcephalum*
+	Pedicels 5–10 mm; fruit ellipsoid; mericarp near round in cross section (Shaanxi)	*Bupleurum dielsianum*
39(34)	Leaves margin white cartilaginous (Sichuan).	*Bupleurum chaishoui*
+	Leaves margin not white cartilaginous.	(40)
40(39)	Fruit vittae 1 in each furrow, 2 on commissure (Xinjiang).	*Bupleurum exaltatum*
+	Fruit vittae 3 in each furrow, 4 on commissure.	(41)
41(40)	Bracts 1–3, small, linear or squamose; bracteoles shorter than or equaling umbellules (Gansu, Qinghai, Sichuan, Xizang).	*Bupleurum malconense*
+	Bracts 3–8, oblong or narrowly elliptic; bracteoles longer than the umbellules.	(42)
42(41)	Bracts 5–8, narrowly elliptic; rays 4–11, somewhat unequal, 1–2.5 cm (Yunnan).	*Bupleurum kunmingense*
+	Bracts 3–4, oblong; rays 6–13, very unequal, 0.4–3.3 cm (Qinghai).	*Bupleurum qinghaiense*
