# Supplementary material for: Phylogenomic analysis of Bupleurum in Western Sichuan, China, including an overlooked new species
Source: Front Plant Sci. 2023 Nov 27;14:1294670. doi: 10.3389/fpls.2023.1294670 (PMC10715590; doi:10.3389/fpls.2023.1294670)
Supplement: Supplementary File 2 — Key to Chinese Bupleurum (modified from Flora of China) [file DataSheet_2.docx]

**Methods**

**Simple sequence repeats (SSRs) and short dispersed repeats (SDRs) analysis**

MISA was used to search for SSRs (Thiel *et al.*, 2003), with minimum numbers of 10 repeat units for mono-, 5 repeat units for di-, 4 repeat units for tri-, and 3 repeat units for tetra-, penta-, and hexa-nucleotide SSRs. SDRs were analysed using REPuter (Kurtz *et al.*, 2001), including forward, reverse, complement and palindromic, with the minimum repeat size being set to 30 bp or more and the Hamming distance being set to 3.

**Nonsynonymous (Ka) and synonymous (Ks) substitution rates**

Each gene in 80 PCGs from the six species was aligned using MAFFT v1.3.7, and then KaKs Calculator v2.0 (Wang *et al.*, 2010) was used to calculate nonsynonymous (Ka) and synonymous (Ks) substitution rates. Ka/Ks values were estimated using the method described by Yang & Nielsen (Yang and Nielsen, 2000) to analyze the selection pressure on each gene. Furthermore, a super matrix composed of concatenated PCG sequences of every samples were constructed to calculate Ka and Ks within and between species. When there were no Ks or very low Ks values in the comparison sequence and a non-computable (NA) value occurred, the Ka/Ks value was manually changed to 0.

**Results**

**Simple sequence repeats (SSRs) and short dispersed repeats (SDRs)**

Analysis of SSRs showed that the total number of SSR sites ranged from 56 (*B. microcephalum*) to 68 (*B. chaishoui*). the distribution pattern of SSRs was similar in the six *Bupleurum* species, with mono-nucleotides being the most common among SSRs (62.9-71.4%), followed by di-nucleotides (6.8-14.5%). Tri-nucleotides (7.9-11.7%) and tetra-nucleotides (6.3-11.9%) were similar, with penta-nucleotides (0-4.8%) and hexa-nucleotides being the least frequent (0-1.7%) (Figure S3-A). Analysis of SDRs showed that the total number of SDRs for the six *Bupleurum* species chloroplast sequences ranged from 40 (*B. chaishoui*) to 49 (*B. microcephalum*), with more forward and palindromic repeats and a minimal number of reverse and complement repeats. (Figure S3-B).

**Nonsynonymous (Ka) and synonymous (Ks) substitution rates and selective pressure**

The synonymous and nonsynonymous substitution ratios (Ka/Ks) were calculated separately for the 80 protein-coding genes of the chloroplast genomes of the six *Bupleurum* species. Most genes had greater synonymous mutation values than nonsynonymous mutation values (Figure S7-A), the Ka/Ks values of all PCGs were less than 1 (Figure S7-B). Among these genes, *ycf*15 had the highest Ka/Ks ratio (0.51), followed by *ndh*D (0.49), *mat*K (0.45), *ycf*2 (0.43); genes with Ka/Ks ratio between 0.3~0.4 were *rps*16, *rpl*22, *ccs*A, *acc*D, *rpo*C2, and *rps*4. The Ka/Ks ratios of the remaining genes ranged from 0 to 0.3. The mean Ka/Ks ratios of protein-coding genes in LSC (0.11) and SSC regions (0.16) were lower than those in the IR regions (0.47). After functional classification and grouping of PCGs, the mean Ka/Ks values of photosynthetic PCGs (0.09) were clearly lower than those of self-replicating PCGs (0.16) and other PCGs (0.23).

At the species level, the selection pressure among *Bupleurum* species was assessed by linking all 80 genes into a supermatrix. The Ka/Ks ratios among the six *Bupleurum* species ranged from 0.19 to 0.31, with an average ratio of 0.22 (Figure S7-C). The highest of all Ka/Ks ratios within species was *B. wenchuanense* (0.37) and the lowest was *B. pseudochaishoui* (0).

The substitution pattern (Ka and Ks, and Ka/Ks ratio) is a well-recognized marker for understanding the evolutionary forces shaping chloroplast genes, reflecting selective pressure on genes. Ka/Ks >1, =1 and Ka/Ks <1 represents positive, neutral and purifying selection, respectively (Guo *et al.* 2017; Yang and Nielsen 2000). In our analysis, all genes had Ka/Ks values less than 1, and synonymous mutations occurred more frequently than non-synonymous mutations in most genes, indicating strong purifying selection on genes. Purifying selection is one of the most common mechanisms of natural selection that continuously eliminates harmful mutations; most of the genes in the cp genomes of six *Bupleurum* species retain their conserved functions (Wu *et al.* 2020; Yang *et al.* 2005). In a previous study of ours on four endemic alpine *Bupleurum* species in Southwestern China, four genes (*mat*K, *ycf*2, *acc*D and *clp*P) were found subjected to positive selection (Huang *et al.*, 2021a). The difference may be related to their different habitats, and the diversity of habitat types may help to increase mutation rates. The four alpine *Bupleurum* species grow in open forests and grasslands on wet slopes, whereas the six species of *Bupleurum* in this study grow in drier environments similar to those species growing in grasslands in northern part of China, such as *B. chinense* and *B. scorzonerifolium*, etc., possibly result such a difference in pressure selection (Huang *et al.* 2021a, He *et al.* 2015, Lu *et al.* 2018). Furthermore, the habitats of the six species are of almost the same environment conditions, the Ka/Ks values within species and between species were also rather low. This is quite different to the species distributed in other regions (Huang *et al.*, 2021a).

**References**

Guo, Y., Liu, J., Zhang, J.F., Liu, S.Y. and Du, J.C. (2017). Selective modes determine evolutionary rates, gene compactness and expression patterns in *Brassica. The Plant Journal,* **91**(1), 34–44.

He, K., Hu, N.Q., Chen, X., Li, J.T. and Jiang, X.L. (2015). Interglacial refugia preserved high genetic diversity of the Chinese mole shrew in the mountains of Southwest China. *Heredity,* **116**(1), 23–32.

Kurtz, S. (2001). REPuter: the manifold applications of repeat analysis on a 620 genomic scale. *Nucleic Acids Research,* **29**(22), 4633–4642.

Lu, L.M., Mao, L.F., Yang, T., Ye, J.F., Liu, B., Li, H.L., Sun, M., Miller, J.M., Mathews, S., Hu, H.H., Niu, Y.T., Peng, D.X., Chen, Y.H., Smith, S.M., Chen, M., Xiang, K.L., Le, C.H., Dang, V.A., Lu, A.M., Soltis, P.S., Soltis, D.E., Li, J. and Chen, Z.D. (2018). Evolutionary history of the angiosperm flora of China. *Nature*, **554**(7691), 666 234–238.

Thiel, T., Michalek, W., Varshney, R.K. and Graner, A. (2003). Exploiting EST databases for the development and characterization of gene-derived SSR-markers in barley (*Hordeum vulgare* L.). *Theoretical and Applied Genetics*, **106**(3), 411–422.

Wang, D.P., Zhang, Y.B., Zhang, Z., Zhu, J. and Yu, J. (2010). KaKs_Calculator 2.0: A toolkit incorporating Gamma-Series methods and sliding window strategies. *Genomics, Proteomics and Bioinformatics*, **8**(1), 77–80.

Wu, Z.H., Liao, R., Yang, T.G., Dong, X., Lan, D.Q., Qin, R. and Liu, H. (2020). Analysis of six chloroplast genomes provides insight into the evolution of *Chrysosplenium* (Saxifragaceae), *BMC Genomics*, **21**(1), 621.

Yang, Z.H. and Nielsen, R. (2000). Estimating synonymous and nonsynonymous substitution rates under realistic evolutionary models. *Molecular Biology and Evolution*, **17**(1), 32–43.

Yang, Z.H., Wong, W.W.S. and Nielsen, R. (2005). Bayes empirical Bayes inference of amino acid sites under positive selection. *Molecular Biology and Evolution*, **22**(4), 1107–1118.

**Figure**

Figure S1. Simple sequence repeats (SSRs) and short dispersed repeats (SDRs) in the six Bupleurum species

(A)SSRs; (B) SDRs

Figure S2. Selective pressure in six Bupleurum species

(A) Ka and Ks rata of 80 protein-coding genes; (B) Ka/Ks ratios of 80 protein-coding genes; (C) Pairwise Ka/Ks ratios among six Bupleurum

Figure S3. Phylogenetic trees based on the highly-variable regions in cp genome.

(A)rpl32-trn; (B) petA-psbJ; (C) trnK-rps16; (D) ycf; (E) combined analysis of sequences rpl32-trnL, trnK-rps16 and petA-psbJ; (F) combined analysis of sequences rpl32-trnL, trnK-rps16, petA-psbJ and ycf1.
